# Polarization sensitive optical coherence tomography with single input for imaging depth-resolved collagen organizations

**DOI:** 10.1038/s41377-021-00679-3

**Published:** 2021-11-24

**Authors:** Peijun Tang, Mitchell A. Kirby, Nhan Le, Yuandong Li, Nicole Zeinstra, G. Nina Lu, Charles E. Murry, Ying Zheng, Ruikang K. Wang

**Affiliations:** 1 Department of Bioengineering, University of Washington, 3720 15th Ave NE, Seattle, WA 98195 USA; 2 Department of Otolaryngology- Head and Neck Surgery, Facial Plastic and Reconstructive Surgery, University of Washington, Seattle, WA 98195 USA; 3 Department of Pathology, University of Washington, Seattle, WA 98109 USA; 4 Center for Cardiovascular Biology, University of Washington, Seattle, WA 98109 USA; 5 Institute for Stem Cell & Regenerative Medicine, University of Washington, Seattle, WA 98109 USA; 6 Department of Ophthalmology, University of Washington, Seattle, WA 98105 USA

**Keywords:** Imaging and sensing, Biophotonics

## Abstract

Collagen organization plays an important role in maintaining structural integrity and determining tissue function. Polarization-sensitive optical coherence tomography (PSOCT) is a promising noninvasive three-dimensional imaging tool for mapping collagen organization in vivo. While PSOCT systems with multiple polarization inputs have demonstrated the ability to visualize depth-resolved collagen organization, systems, which use a single input polarization state have not yet demonstrated sufficient reconstruction quality. Herein we describe a PSOCT based polarization state transmission model that reveals the depth-dependent polarization state evolution of light backscattered within a birefringent sample. Based on this model, we propose a polarization state tracing method that relies on a discrete differential geometric analysis of the evolution of the polarization state in depth along the Poincare sphere for depth-resolved birefringent imaging using only one single input polarization state. We demonstrate the ability of this method to visualize depth-resolved myocardial architecture in both healthy and infarcted rodent hearts (ex vivo) and collagen structures responsible for skin tension lines at various anatomical locations on the face of a healthy human volunteer (in vivo).

## Abstract (web summary)

Discrete differential geometry-based polarization tracing method enables PSOCT imaging of depth-resolved collagen organizations.

## Graphical abstract

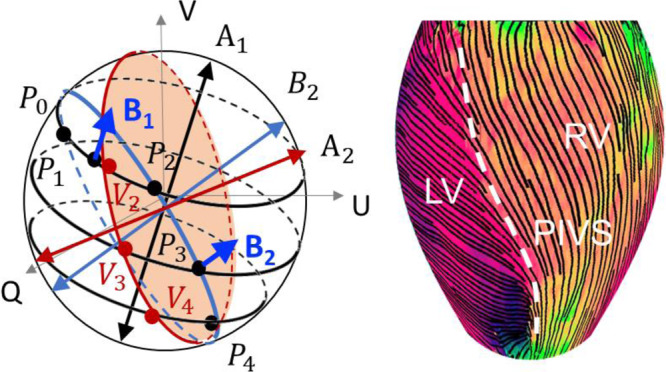

## Introduction

Collagen, often organized in the form of fibers, is a structural protein widely distributed in biological tissue^1^. Its organization plays an important role in biomechanical structure and function. For example, the alignment of dermal collagen contributes to anisotropic tensile strength in human skin tissue (related to Relaxed Skin Tension Lines (RSTLs)) which provides both protection and tailored flexibility^2,3^. Collagen organization also reveals important physiologic function in tissue such as the heart, where its organization contributes to unique contractile muscular behavior^4,5^. In cases where collagen networks have been disrupted, such as after myocardial infarction, subsequent functional decline has been documented^4,5^. Such examples suggest that insights into biological and pathological structure-function relationships may be elucidated, and even used to drive clinical intervention, based on depth-resolved identification of collagen fiber organization.

While techniques, such as polarized light microscopy, have been utilized to image collagen fibers in an ex vivo setting, many require tissue sectioning procedures^6,7^. To achieve in vivo collagen imaging, techniques such as second harmonic generation (SHG) confocal-type microscopy have been developed to visualize and analyze depth-resolved collagen organization^8–11^. However, the field of view (FOV) (up to ~500×500 µm^2^) and imaging depth (up to ~200 µm) are often too limited to reconstruct macroscopic collagen organizations that reveal the functional architecture of the tissue. Clearly, a technique capable of wide-field three-dimensional (3D) imaging of collagen structures in living tissue would provide rich information on how collagen organization relates to physiologic function.

Polarization-sensitive optical coherence tomography (PSOCT)^11–16^ is a promising technique that enables noninvasive 3D mapping of birefringent material. Since fibrous collagen is highly birefringent, PSOCT can contrast collagen structure with a wide FOV (from a few to tens of square centimeters), relatively deep imaging depth (up to ~2 mm) and fast imaging speed (seconds per 3D scan)^17–20^. However, traditional PSOCT provides only accumulated polarization information along depth due to that back-scattered light must pass through tissue twice before detection. Such accumulation can make it difficult to interpret the birefringent properties of the sample.

An elegant Jones matrix-based algorithm was developed to map local axis orientation using a single incident polarization state^21^, in which local axis orientation is considered as a scalar constrained within *QU*-plane. However, the asymmetry in the imaging system (i.e., the optical path in the system is not in round-trip) can cause an overall rotation of the optic axes away from the *QU*-plane^14,22–24^. That is, the local axis orientation to be measured becomes a 3D vector in Stokes space. As such, the algorithm would result in inaccurate determination of the local axis orientation and local phase retardation over depth.

The 3D local axis orientation in Stokes space can also be achieved by using two distinct incident polarization states in the measurements^14,18,25^, which can practically be realized by additional hardware modules that, however, reduce the imaging range by half^11,19^, or are expensive, and require additional hardware control which can complicate the imaging system setup^25–27^.

Rather than using the Jones matrix model to describe the birefringent property, here we propose a PSOCT-based polarization state transmission model that leverages the Poincare sphere to describe the evolving polarization state of an incident beam as it travels through a birefringent sample with depth-varying optic axes and phase retardation in a round-trip measurement. Based on this transmission model, we describe a novel method where the 3D local axis orientation in the Stokes space can be obtained with only one single input polarization state, giving depth-resolved collagen imaging with sufficient imaging quality. The technical challenges of resolving birefringent information (i.e., local phase retardation and 3D local axis orientation in the Stokes space) along the depth with a single input polarization are addressed using a discrete differential geometry (DDG) based polarization state tracing (PST) method combined with a series of 3D rotation operations in the transmission model. Without the requirement of multiple input polarization states, this method can simplify the imaging system setup and mitigate motion artifact, being aimed for clinical translation.

Using this method, we demonstrate depth-resolved mapping of the birefringent properties (largely determined by collagen organization) in ex vivo rodent hearts and in healthy human skin, in vivo. For the ex vivo study, fiber disorganization is detected within an infarct rodent heart using the proposed depth-resolved collagen organization imaging method. For the in vivo study, we demonstrate depth-resolved collagen organization imaging in healthy human skin to reveal the facial skin tension lines.

## Results

To validate and demonstrate the usefulness of the proposed method, we first established a swept source PSOCT system (Fig. S1) operating at 1310 nm wavelength with an axial resolution of 7.5 μm in air (See Supplementary Section S1). With this system, the proposed DDG-based PST method was validated using a custom-made thin strip phantom of polylactic acid (PLA) 3D printer filaments, which exhibit intrinsic homogeneous birefringence with its fast optic axis parallel to the filament’s axis^28^ (see Supplementary Section S2). The measured optic axis maps agreed with expected values (Figs. S2 and S3), demonstrating the validity of the proposed method to derive depth-resolved relative optic axis orientation.

Since fibrous collagen shows intrinsic optical anisotropy, axis orientation mapping can be utilized to extract the collagen organization from the tissue with high contrast and provide orientation information of collagen alignment^29–31^. Next, we demonstrate depth-resolved collagen organization imaging of several samples of importance using the proposed method. In all collagen orientation results below, we used the same colormap (Supplementary Section S2) to avoid possible confusion. The 0 deg is defined as the sample orientation when it is parallel to the B-scan direction (i.e., fast scanning direction). To reduce noise, a spatial averaging filter (4 × 4 pixels, x × y) was applied to the en-face and the cross-sectional (x × z) optic axis orientation images.

### Ex-vivo imaging of healthy mouse heart

**Fig. 1:**
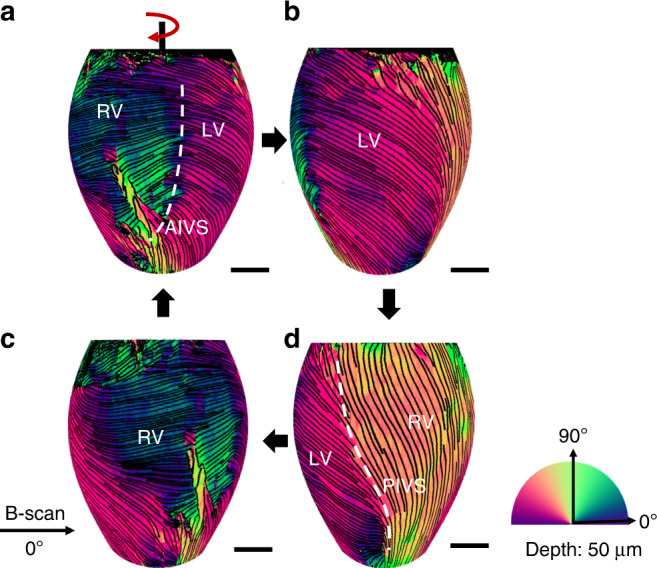
Myocardial fiber orientation mapping of a whole mouse heart. With the proposed DDG-based PST method, single input PSOCT is capable of providing the orientation information of myocardial fiber within superficial ventricular wall of a whole mouse heart. Results shown are different perspective views of a reconstructed 3D optic axis orientation map at 50 μm below the heart surface of a whole heart (Supplementary Section S3). **a** The front view; **b** The right-side view to show left ventricle; **c** The left-side view to show right ventricle; **d** The back view of the 3D volume-rendered myocardial fiber organizations in the whole heart. The colormap is shown in the lower right corner. The streamlines on the heart represent the orientation of the myofibers. In the lower left corner, the black arrow indicates the B-scan direction, which is defined as the 0°. The scale bar = 1 mm. RV right ventricle, LV left ventricle, AIVS anterior interventricular sulcus, and PIVS posterior interventricular sulcus

To demonstrate the ability of the DDG-based PST method to visualize collagen organization, a whole mouse heart was imaged by the PSOCT system with a lateral resolution of 22 μm (details see Supplementary Section S3) within 1 h of extraction. Figure 1 shows a 3D rendered PSOCT volume of the whole heart obtained from stitching separate en-face axis orientation images (50 μm below the heart surface) acquired at 12 perspective angles (Supplementary Section S3). In this image, streamlines obtained by the local axis orientation values are used to represent the fiber tracts and show the skeleton structures in the spatial organization of fiber bundles. The result depicts the myocardial fiber organization of the superficial tissue of the whole mouse heart, where a typical helical-like myocardial fiber structure was reconstructed by the DDG-based PST method, demonstrating the ability of the proposed method to reconstruct myocardial fiber orientations.

Taking advantages of the wide FOV of the current PSOCT system, the relative fiber orientations among different parts of the heart were visualized in a macroscopic view. The myocardial fiber organization of left ventricle (LV) (Figs. 1a, 1b, and 1d), right ventricle (RV) (Figs. 1a, 1c and 1d) and the two boundaries between the RV and LV: the anterior interventricular sulcus (AIVS) (white dash line in Fig. 1a) and the posterior interventricular sulcus (white dash line in PIVS) (Fig. 1d) are presented in an entirety in Fig. 1. The results show the potential for this technique to investigate microstructural foundations which may play a role in the interaction mechanisms between different cardiac tissues.

### Ex-vivo imaging of infarct rat heart

Herein we demonstrate the ability of the DDG-based PST method to image collagen orientation and organization in both healthy cardiac tissue and in tissue 4 weeks following an induced infarct event (see Supplementary Section S4). Briefly, one animal was randomly chosen as the healthy sample, and another chosen to undergo a thoracotomy surgery for ischemia/reperfusion of the left anterior descending coronary artery to create a myocardial infarction. Following euthanasia, the aortas of both samples were fixated using 4% paraformaldehyde perfusion prior to PSOCT imaging.

**Fig. 2:**
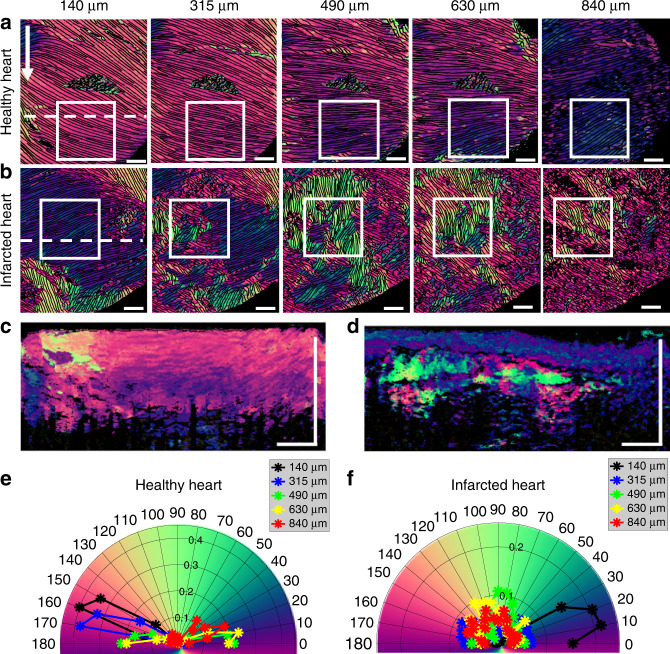
Depth-resolved myofiber organizations in healthy and infarcted rat hearts. With the proposed DDG-based PST method, single input PSOCT is capable of imaging the depth-resolved myofiber disorganization in infarct rat hearts. Results shown were obtained from healthy and infarcted hearts, respectively. The en-face local axis orientation (OAx) images of **a** the healthy rat heart **b** the infarct heart at different depths as shown from 140 μm to 840 μm. Cross-sectional axis orientation images of **c** the healthy rat heart **d** the infarct heart at the regions indicated by the white dash lines in **a** and **b**. **e, f** The polar histogram distributions of orientation obtained from the white boxes in the healthy heart **a** and the infarct heart **b** at five depths relative to the tissue surface (140 μm, 315 μm, 490 μm, 630 μm and 840 μm), respectively. White arrow at the top left points to the apex. The yellow arrow indicates the B-scan direction, which is defined as the 0°. Note that a triangular black tape was used as the guide to align the system scanning during imaging, which appears as an artifact in the middle of images in **a**. The scale bar = 1 mm

Linear gradient changes in the fiber orientation angle along depth was detected in the healthy heart (Figs. 2a, 2c and 2e), consistent with prior histology studies^32,33^. In the en-face images (50 μm below the surface) (Fig. 2a) selected at different depths (from 140 to 840 μm relative to tissue surface), the myofiber orientations were highly uniform at each depth and rotated gradually: the mean axis orientation determined from each en-face slice was 159.7° (i.e., −20.3°) ± 2.35°, 167.2° (i.e., −12.8°) ± 2.30°, 4.19° ( ± 2.49°), 11.7° ( ± 3.45°) and 45.7° ( ± 4.51°), respectively, with relative small standard deviations. This phenomenon is displayed using a polar histogram plot to visualize the dominant orientation direction and relative distribution of the optic axis at each depth (Fig. 2e). The preferential collagen distribution demonstrates myofibril uniformity and anticlockwise rotation of the dominant fibers along depth. Depth-resolved reconstruction shows the gradient change of the myofibril direction over depth, consistent with the prior histological observations^32,33^, demonstrating the usefulness of the proposed DDG-based PST method.

However, the infarcted heart exhibited disorganization of the myocardial fibers along the depth within the cardiac wall (Figs. 2b, 2d and 2f). In the en-face axis orientation images selected at different depths (Fig. 2b), myofibers in the superficial layer (140 μm relative to the tissue surface) appear uniform, which is similar to the healthy heart (e.g. Figure 2a). With the increase of depth, the fiber orientations show random and discontinuous patterns in the infarcted tissue (Fig. 2b), indicating fibril damage due to the infarct occurred relatively deep within the cardiac wall. The cross-sectional image (Fig. 2d) shows that the collagen fiber disorder begins around 300 μm from the outer wall. Beneath this depth, a linear gradient change in fiber orientation is no longer observed, demonstrating remodeling of the fiber structure after infarction. The orientation distribution of the fibers at each depth in the infarct tissue is shown in the polar histogram (Fig. 2f), where except for the surface layer (140 μm), the distributions of the orientation in the infarct tissue are widely spread in the deeper layers. These results are consistent with histological staining (Fig. S5b), where the superficial tissue of the infarcted heart appears normal (similar to the tissue of the healthy heart (Fig. S5a)) while random fiber structures appear around 300 μm in depth.

Depth-resolved axis orientation images of healthy and infarcted rat hearts revealed complex myocardial fiber arrangements which differ along the depth of the cardiac wall. The depth-resolved ability of the DDG-based PST method detected physiologic change deep within the cardiac wall. Overall, rodent heart imaging in this work demonstrated that the method can be readily applied to laboratory studies, potentially providing an insight into the physiology and pathology of the biological tissue.

### In-vivo imaging of human skin

**Fig. 3:**
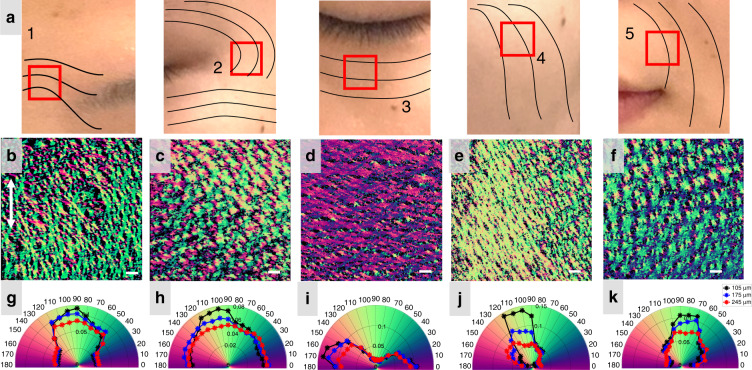
Collagen organizations at various locations on the human facial skin. With the DDG-based PST method, PSOCT reveals macroscopic architecture of collagen organizations at various locations on the human facial skin. **a** Illustration of selected regions (marked as red squares, 1–5) on the human facial skin for representative PSOCT imaging of the relaxed skin tension lines (RSTLs), where 1–5, respectively indicate the facial regions of glabella, lateral eyelid, inferior eyelid, cheek, and upper lip. **b–f** The en-face axis orientation (OAx) PSOCT images (generated by average projection of a 70 μm slab, starting from approximately 105–175 μm depth from the skin surface) acquired from the regions of **b** glabella; **c** Lateral eyelid; **d** Inferior eyelid; **e** cheek; **f** upper lip. **g–k** The corresponding histogram distributions of orientation obtained from the en-face OAx slices measured at **g** glabella; **h** lateral eyelid; **i** inferior eyelid; **j** eye bag; **k** upper lip, respectively, at three different depths (105 μm, 175 μm and 245 μm). White double-headed arrow in (B) shows the direction of the body axis. The yellow arrow indicates the B-scan direction, which is defined as the 0°. The scale bar = 500 μm

Relaxed skin tension lines (RSTLs), corresponding to the natural orientation of elastin and collagen fibers, are important for planning and placement of surgical incisions in facial reconstructive surgery and cosmetology. Because the relative angle between RSTLs and incisions plays a role in the formation of scars^2,3^, visualizing fiber orientation in human skin tissue may provide surgeons with additional information to both personalize surgical procedures and monitor long term outcomes. To demonstrate the potential of the DDG-based PST method in surgical applications, we imaged multiple anatomical locations (defined according to aesthetic units shown in Fig. 3a) on the face of a healthy adult volunteer (29 years, female, Asian) (see Supplementary Section S5). Two OCT scanning patterns were used for imaging: high-lateral resolution scanning (11 μm) with a field of view (FOV) of 2 mm×2 mm, and relatively low-lateral resolution scanning (22 μm) with a FOV of 6 mm×6 mm. RSTLs, defined as the direction of greatest tension on the skin and parallel with collagen bundles^34–36^, were estimated and displayed on the photograph (Fig. 3a) for reference^2,34^.

Woven mesh-like structures formed by varied arrays of collagen bundles were observed in the en-face axis orientation images (Fig.3c–g, produced by depth-averaging a 70 µm thick slab with its anterior boundary at 100 µm beneath the skin surface) in each facial region, demonstrating that collagen organization of the skin can be extracted from living tissue using the proposed technique. In general, a single dominant color was found in the en-face image of each facial region, suggesting alignment of collagen fibers which can be measured and visualized using the DDG-based PST method. Polar histograms (Fig. 3h–k) which present orientation distribution in the en-face image at each depth show that the preferential direction of the collagen fibers is consistent with the general directions of RSTLs as shown in Fig. 3a. We note that the collagen orientation of glabella (Fig. 3g) is orthogonal to the direction of traditional RSTLs. While this may be a feature of the particular volunteer, there remains unanswered questions regarding the relationship between collagen fiber alignment and RSTLs.

These results demonstrate the potential for the DDG-based PST method to guide skin incision placement by visualization patient-specific RSTLs in vivo, further investigate collagen fiber orientation in normal and abnormal states of scar formation, and study collagen disorders of the skin such as Ehler’s Danlos syndrome and Scleroderma^37,38^.

**Fig. 4:**
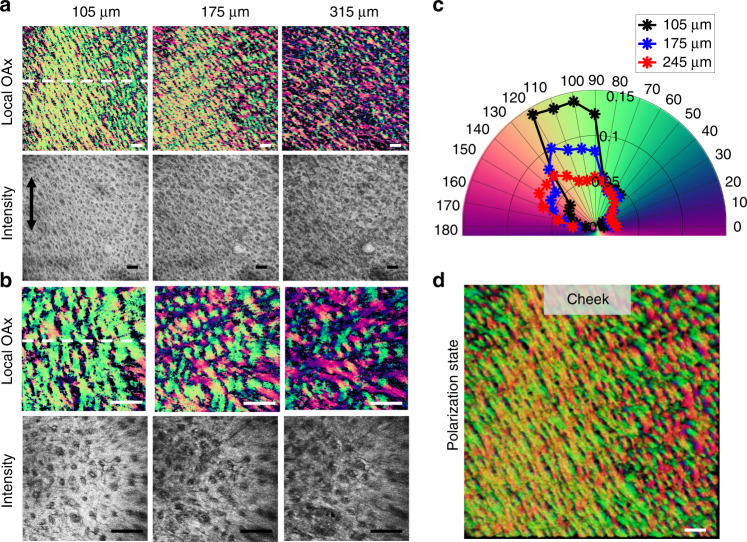
Depth-resolved collagen organizations within the cheek skin. Depth-resolved collagen fiber orientations within facial skin are revealed by PSOCT images. Results shown are obtained from a selected region of cheek skin (marked as red square in Fig. 3a). **a, b** The en-face local optical axis orientation (OAx) images and the corresponding en-face OCT intensity images at three different depths (105, 175, and 245 μm) using high and low lateral resolution scanning probe, respectively; Black double-headed arrow shows the direction of the body axis. **c** The histogram distributions of orientation obtained from the en-face OAx slices shown in **a** (105, 175, and 245 μm, respectively). **d** 3D polarization state image. The scale bar = 500 μm. The yellow arrow indicates the B-scan direction, which is defined as the 0°. Please also see the PSOCT movies showing axis orientations at all depths: cheek (LSM 03).mov and cheek (LSM 02).mov

The DDG-based PST method was also used to image depth-resolved features of in vivo facial collagen organization. Two scanning patterns (see Supplementary S5) are used to reveal networks of collagen bundles at different FOVs in the cheek across all depths (Fig. 4a, b). The mesh-like structure of collagen organization is clearly presented in the local axis orientation images while crossing networks are faintly perceivable in corresponding structural en-face images (obtained by OCT images). In the axis orientation images (Fig. 4a, b), the collagen fibers in the papillary layer (~105 μm) appear to form a loosened mesh but with an alignment whose orientation is matched with the RSTLs. Multiple arrays of collagen fibers with different orientations are found to form a tight meshwork in the reticular layer (~175 μm and 245 μm).

The polarization state image (Fig. 4d) was created using the output polarization states^12^ where red, green and blue are used to code the Stokes parameters Q, U and V (respectively) corresponding to the output polarization state in backscattered light. Since the polarization state image reveals comprehensive information (i.e., combining both phase retardation and orientation) of the birefringent sample^12^, it can visualize the birefringent component embedded within the tissue with high contrast. The top view of the 3D polarization state image of facial skin (cheek) is provided to visualize the architecture of the collagen fibers as shown in Fig. 4d, the colormap of which is the color-coded Poincare sphere in Fig. S1b–d. Mesh-like woven organization of the collagen fibers are clearly seen. Polarization results taken on other facial areas can be found in the Supplementary Materials (Figs. S6–S9), as well as videos.

These results demonstrate the potential of the proposed method to investigate 3D collagen structure in both macro and micro-scale, potentially connecting how collagen organization maintains the structural integrity of the skin.

## Discussion

Clinical translation of PSOCT for depth-resolved imaging of fiber orientations for in vivo imaging requires minimal system complexity, cost-effectiveness and flexibility. The DDG-based PST approach introduced here enables depth-resolved birefringent imaging that only requires one single input polarization state, allowing a generalizable single input PSOCT system setup. Differing from previous multiple input Jones matrix-based measurement^25,26^, the DDG-based PST approach analyzes the trajectory of the depth evolution of a single input polarization state in the Stokes space.

Due to the inevitable asymmetric property of the imaging system, the optic axis to be measured becomes a 3D vector in the Stokes space rather than a scalar constrained within the *QU*-plane as described in^22^. This is because the polarization states scattered back from the sample are routed to the detector that takes a different optical path from the input light being delivered to the sample, leading to a system-induced change in the detected polarization states. The change can be considered as a global rotation to the output polarization states on the Poincare sphere. Based on the rotation model that describes the relation between the evolution of the output polarization states and the optic axis, the measured optic axis becomes a 3D vector in the Stokes space. Since the global rotation would not alter the relative geometrical relationship between the polarization state vectors scattered back from the sample, the relative orientation among the measured optic axes using the proposed PST method can still reveal the relative orientation among the true optic axes within the sample. Hence, compared with the previous work that using a single input polarization state to derive the local phase retardation and local axis orientation in the 2D *QU*-plane^21^, the DDG-based PST approach has an advantage that it can improve the measurement accuracy through determining the local axis orientation in 3D Stokes space (see Supplementary section 6).

The advantage of the DDG-based PST approach over the previous methods that use two input polarization states is that without introducing the additional polarization modulation scheme^24,27,39^, the requirement of known polarization states incident on the sample can be removed. This is because the DDG-based analysis of the trajectory of the polarization states is not affected by the location of the start point (i.e., the values of the input polarization state). Particularly, for any polarized light (excluding the one parallel to the optic axis of the sample) transmitting through the same region of the sample, its corresponding trajectories of the polarization evolution on the Poincare sphere should share the same set of binormal vectors, which determines the unique local birefringent parameters. This theoretical conclusion also provides a potential ability of the proposed method to relax the requirement for other PSOCT configurations, which is particularly attractive to the efforts that aim for clinical translation.

Another benefit of the proposed method is that it makes it compatible with most PSOCT system designs which utilize a single input polarization state, providing flexibility in system design. Previous studies have shown that different input polarization states have different sensitivities at particular values of optic axis or retardance^40^. Therefore, a general design with flexible selection of the input polarization state^40^ is desirable for achieving the optimal sensitivity when imaging different birefringent samples.

However, there remains an issue to be addressed in the proposed method. It is noted that a transition from one layer to another layer with a distinct difference in optic axis, would result in a false binormal vector at the transition position that is not accurate for any single layer. In this case, the binormal vector is instead an integrated result from both layers. This error can propagate and accumulate when computing the local axis orientation over depth. Because the binormal vector at the transition position does not belong to the upper layer, nor the lower layer, it can be considered as a jitter that can be recognized by the differential method. By removing such points, the estimation error introduced by the interface between different birefringent components can be mitigated.

In most cardiac tissue studies, histology remains the gold-standard method to evaluate myofiber architectures. However, this method is destructive and time consuming. The ability to reconstruct 3D collagen architecture from 2D stacks of histological sections is also challenging. Notably, rat heart imaging was performed following paraformaldehyde fixation which has been known to stiffen tissue based on the induction of collagen cross-links. However, the general helical-like arrangement of collagen tissue appeared to be retained in the healthy heart.

In this study, we note that 22 μm lateral resolution may not be sufficient for detecting minute changes in collagen organization. Also, it is difficult to observe the dynamics of the myocardial fibers of the whole heart during heart beating because there is a time delay induced by the point scanning protocol when imaging different parts of heart. These two issues can be mitigated simultaneously by integrating the full-field microscopy into the PSOCT system. A high-resolution (~1 μm) 3D volumetric collagen organization image can be obtained with a single shot rather than point scanning. However, imaging depth would decrease in this case.

Overall, the depth-resolved PSOCT images of myofibers may be further used to monitor healing response to an infarct event at various times from injury, or in remodeling of grafts in heart, for example. The understanding of these matrices and remodeling could help understanding many important physiological diseases, such as biomechanical origin of arrhythmia etc.

In human facial skin, several aesthetic units were selected to be imaged using PSOCT to show their corresponding collagen organizations. These regions include the thinnest facial skin (eyelid) to the thickest facial skin (glabella) and demonstrate applicability of PSOCT throughout facial skin. These are also the areas commonly affected by skin cancer, which often requires surgical resection and reconstruction with tissue flaps. Minimizing wound tension and orienting incisions along skin tension lines is clinically critical in optimal scar healing reconstruction of facial defects.

Numerous methods describing skin tension lines exist. One of the earliest methods is Langer’s Lines, based on the natural elliptical tendency of circular wounds created in a cadaver^2^. The most commonly employed method in modern surgery is the Relaxed Skin Tension Lines, determined by pinching skin and observing furrows in the living body^3^. No available method has been formulated with objective knowledge of the collagen fiber organizations. The method proposed in this study enables fast, in-vivo, non-invasive, wide field and depth-resolved collagen orientation imaging, providing an additional metric to give insight into collagen organizations, which make up RSTLs in each individual living subject.

While we have demonstrated that the DDG-based PST method can be used to extract depth-resolved collagen organization using the single input PSOCT system, further improvement is required. We neglected the dichroism (i.e., the anisotropic scattering attenuation of incident light)^16^ of the sample in our current treatment. As the attenuation is one of important parameters in some ophthalmologic studies to characterize the features of the diseases^41^, further improvement is required if the diattenuation property of the sample is of concern. Previous studies have shown that the attenuation of the sample can twist the circular curve out of its osculating plane, and hence the trajectory of the output polarization states would become a spiral curve rather than a circular curve at the Poincare sphere^42^. One potential solution to evaluate the attenuation property of the sample is to calculate the torsion value of the trajectory curve and then obtain the degree of the curve twisting out of the plane of the curvature. The bigger the absolute value of the torsion is, the stronger the attenuation would be. In addition, multiple scattering in turbid tissue is known to limit polarization imaging depth and contrast when it is not being considered in the model formulation. Optical clearing^43–45^, a technique that can reduce scattering in the skin and is biologically compatible with cosmetic procedures, may be employed together with PSOCT imaging in future studies to increase the probing depth and contrast.

Finally, the simplicity of design in PSOCT and the widespread clinical use of traditional OCT imaging suggest that birefringent information may serve as an additional contrast from which clinicians may pull from to make interventional decisions. OCT has been used clinically for visualizing vascular function in various pathological states^46^, as well as to detect changes in light-scattering properties of fibers associated with the healing process of burn wounds^47^. More recent studies have also introduced non-contact measurements of elasticity using optical coherence elastography, where biomechanical anisotropy has been shown to generally agree with collagen fiber orientation^48^. Together, a non-contact OCT imaging modality coupled with polarization-sensitive measurements has the potential to provide clinicians with multiple contrast mechanisms in a single scan, providing a wealth of diagnostic information in a readily translatable package.

In summary, we expect that the proposed PSOCT technique will enable superior contrast and diagnostic capabilities compared to conventional OCT because of its ability to reveal micro- and macroscopic structural features. Potential clinical applications of PSOCT described here include early detection of acute myocardial infarction, evaluation of scar formation and the imaging-guided plastic surgery. In addition, the significant simplification of the PSOCT imaging system facilitates the clinical translation of the PSOCT imaging technique, which may provide a new matrix of diagnostic information.

## Material and methods

### Discrete differential geometry-based PST method

In Stokes space, a linear polarization can be modeled as a 3D rotation, where the rotation axis represents the optic axis, and the amount of rotation is the degree of phase retardation^23^. That is, when a polarized light propagates through a multi-layered material with a constant optic axis (i.e. material with a constant optic axis but experiencing phase retardation), the evolutional trajectory of the polarization state of the light beam must form a spatial circular curve on the Poincaré sphere. The osculating plane of this circular curve is the rotation plane of the linear polarization. Once this osculating plane is determined, the rotation information, i.e., both the rotation axis representing the optic axis and the rotation angle representing the phase retardation, can be determined. Hence, determining the osculating plane of the curve formed by the evolutional trajectory of the polarization state is the key to derive the local optic axis and phase retardation of a multi-layered material.

One such method to determine the osculating plane is based on discrete-differential geometry (DDG). The DDG analysis computes a set of ‘*TNB*’ orthonormal bases in 3D space where *T* is the tangent vector, *N* is the normal vector and *B* is the binormal vector of the spatial curve which are then used to determine the osculating plane (i.e., the plane that *T* and *N* vectors lie) at each point of the curve^28^. Since PSOCT is based on a round-trip measurement, the measured osculating plane is the apparent plane defined by the accumulated polarization rather than the actual osculating plane.

To retrieve local birefringent information using DDG analysis, a PSOCT-based polarization state transmission model that describes the polarization state evolution in a round-trip measurement is proposed and described herein. This model reveals the relation between local birefringent parameters and the evolution behaviors of the output polarization state in PSOCT. Based on this relationship, the DDG-based PST method is combined with a set of 3D rotation operations to derive the local optic axis and phase retardation from the measurements.

In what follows, we show how the *TNB* vectors are determined using the DDG method on the trajectory of the output polarization states. Then, we show how we utilize the *TNB* vectors to derive the local optic axis and local phase retardation in the PSOCT-based polarization state transmission model, which reveals the relation between local birefringent parameters and the evolution behavior of the output polarization states in the PSOCT system.

In Poincare representation, the trajectory of the output polarization state can be considered as a discrete spatial curve by connecting a sequence of measured data points: $P_n(n = 1,2,3 \ldots )$, where *P*_*n*_ represents the vector of output polarization state as a function of discrete pixel locations, *n*, along the depth. We refer to this discrete spatial curve as the polarization state curve. Hence, a sequence of the right-handed discrete Frenet-frames can be defined and computed by the DDG method as^49^

1 $$K_n = \left( {T_n,N_n,B_n} \right)k = 1,2,3 \ldots$$

where

2 $$\begin{array}{l}T_n = \frac{{P_{n + 1} - P_n}}{{\left| {P_{n + 1} - P_n} \right|}}\\ B_n = \frac{{T_{n - 1} \times T_n}}{{\left| {T_{n - 1} \times T_n} \right|}}\\ N_n = B_n \times T_n\end{array}$$

*T*_*n*_, *N*_*n*_ and *B*_*n*_ are the unit tangent, normal and binormal vectors, respectively. The osculating plane at each point is spanned by the corresponding *T* and *N* vectors. Since *B*_*n*_ is the cross-product between *T*_*n*_ and *N*_*n*_ (follows the right-hand rule), it is orthogonal to the osculating plane and thus includes information related to the optic axis^50^. Note that the osculating plane directly provided by the *TNB* vectors is the apparent osculating plane that not only carries information of the localized tissue, but also includes the accumulated information of all layers on top of it.

**Fig. 5:**
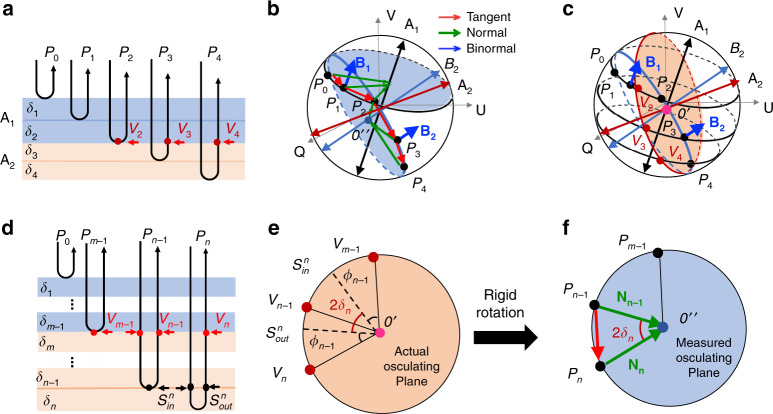
Schematic diagram of the PSOCT-based polarization state transmission model. Shown is the model in a simulated birefringent sample with depth-varying optic axis, representing a A-scan in the imaging. $P_0$, $P_1$, $P_2$, $P_3$ and $P_4$, are the output polarization states that are detected by the PSOCT system. **a** A depth scan of a simulated birefringent sample with depth-varying optics axis, where 4 birefringent layers are simulated with the first 2 layers sharing the same optic axis $A_1$, and the last two layers sharing $A_2$. $V_2$, $V_3$ and $V_4$ are the intermediate polarization state that are only influenced by the local birefringence. **b**The trajectory curve of the output polarization states $P_0$, $P_1$, $P_2$, $P_3$ and $P_4$ on the Poincaré sphere with the corresponding TNB vectors. The measured osculating planes spanned by the T and N vectors are colored by blue. **c** The geometric relation between the upper optic axes, $A_1(i.e.,B_1)$, the axis of the measured osculating plane $B_2$ and the local optic axis $A_2$. The actual osculating plane is colored by red. **d** A depth scan of a simulated birefringent sample with depth-varying optic axis. **e** The actual osculating plane composed by the $V_{{{{\mathrm{m}}}} - 1}$ to $V_{{{\mathrm{n}}}}$. **f** The measured osculating plane composed by $P_{{{{\mathrm{m}}}} - 1}$ to $P_{{{\mathrm{n}}}}$. O’ (pink points) and O” (blue points) are the center of the actual and measured osculating planes, respectively. Red, green and blue arrows represent the tangent (T), normal (N) and binormal (B) vectors, respectively

Next, we describe the PSOCT-based polarization state transmission model, with which the *TNB* vectors are combined with a set of 3D rotation operations to derive the local optic axis of a multi-layered sample with depth-varying optic axes. To illustrate this model, the polarization states of a light beam traveling through a representative birefringent sample is schematically shown in Fig. 5. To simplify, a simulated sample is used with two groups of birefringent material with different optic axes (*A*_1_ and *A*_2_), respectively (Fig. 5a). The first group consists of two retarder layers (blue layer) sharing the optic axis of *A*_1_, whereas the 2nd group also consists of two retarder layers (yellow layer) sharing the optic axis of *A*_2_. When the light beam propagates within this sample, each of the five interfaces back-scatter the incoming light toward the PSOCT detector to form an A-scan and the corresponding output polarization states are $P_0$, *P*_1_, *P*_2_, *P*_3_ and *P*_4_, respectively. The trace of the output polarization state from $P_0$ to *P*_4_ forms a discrete curve on the Poincare sphere surface. DDG computation is then applied to this polarization state curve to determine the *TNB* vectors (red, green and blue vectors represent *T*, *N* and *B* vectors respectively), as shown in Fig. 5b.

Specifically, for light beams scattered back from the sample surface and the first two blue layers, the output polarization states $P_0$, *P*_1_ and *P*_2_ are represented by the black points on the Poincaré sphere (Fig. 5b). Since the first two layers share the same optic axis *A*_1_, the polarization state curve of $P_0$, $P_1$ and $P_2$ on the Poincare sphere must follow a circular curve whose osculating plane is the rotation plane. For these two surface layers, the binormal vector *B*_1_ (which is orthogonal to the osculation plane) is equal to the optic axis *A*_1_ based on the linear polarization rotation model mentioned above:

3 $$A_1 = B_1$$

For output polarization states scattered back from deeper layers with a different optic axis, the corresponding binormal vectors no longer represent the local optic axes. Instead, there is a 3D rotation between the binormal vectors and the exact optic axes in the specific layer of interest. This is why the differential geometry method alone can only determine the optic axis in a homogeneous sample rather than an inhomogeneous sample with varying optic axes (such as the mesh-like collagen fibers in the dermis and the myocardial collagen fibers)^42,51^.

Here, we discuss in detail the 3D rotation relationship among the optic axes, the binormal vectors of the polarization state curve, and the local optic axis of interest, which is required to derive the local optic axis progressively from superficial to deep tissue locations. Figure 5c illustrates the geometric relationship among the upper optic axes by highlighting the transmission process of the polarization states in the sample. On the Poincare sphere, the black points represent measured output polarization states $P_0$, $P_1,P_2$, $P_3$ and $P_4$ while the red points *V*_2_, *V*_3_ and *V*_4_ represent the intermediate polarization states of light when they pass through the interfaces (Fig. 5a). These intermediate states are chosen to be shown because the change in the polarization state from *V*_2_ to *V*_3_ and *V*_2_ to *V*_4_ is only determined by the yellow layers (Fig. 5a), representing the information of the region of interest (ROI) in the tissue without accumulation effect. Hence, the trajectory of these three points must follow along another circular curve on the Poincare sphere, whose binormal vector is equal to the optic axis *A*_2_ of the yellow layers. We call the osculating plane of this curve (determined by *V*_2_, *V*_3_ and *V*_4_) the actual osculating plane. Although the trajectory of *V*_2_, *V*_3_ and *V*_4_ includes pure information of the local axis of ROI, they are the intermediate states which cannot be detected directly due to the round-trip measurement of PSOCT. Instead, they are each modified when light passes through the upper blue layers again to form the resultant output polarization states *P*_2_, *P*_3_ and *P*_4_.

These modifications induced by the double-passage of the backscattered light are the same for all intermediate polarization states (i.e., *V*_2_ to *P*_2_, *V*_3_ to *P*_3_, *V*_4_ to *P*_4_) and can be viewed as a 3D rotation in the Poincare representation because they pass through the same upper blue layers as shown in Fig. 5a. That is, the actual osculating plane of *V*_2_, *V*_3_ and *V*_4_ experiences a 3D rigid rotation about the axis of the upper layers *A*_1_ (by an angle $\delta = \delta _1 + \delta _2$, where $\delta _1$ and $\delta _2$ are the local phase retardation of the first two layers) to form the measured osculating plane of $P_2$, $P_3$ and $P_4$, whose binormal vector $B_2$ can be directly obtained using the DDG method as shown in Fig. 5b. Hence, the local optic axis $A_2$ of the yellow layers can be obtained by rotating the binormal vector $B_2$ about $B_1$ by -$\delta$ as shown in Fig. 5c. This geometric relationship can be expressed as a matrix rotation:

4 $$A_2 = R_1( - \delta ;\;A_1)B_2$$

where $A_1$, $A_2$ and $B_2$ are the 1×3 matrices represented by the corresponding *Q*, *U* and *V* values; $R_1( - \delta ;\;A_1)$ is the 3D rotation matrix determined by $A_1$ and $- \delta$.

Equation 4 can be generalized for the sample with multiple varied optic axes along depth. The actual osculating plane in the layer of interest would experience a set of 3D rotations when the light beam passes through all upper layers again (due to double-passage). Each upper layer would rotate the actual osculating plane once with the rotation matrix determined by the corresponding layer’s local optic axis and local phase retardation. Since the normal vector of the actual osculating plane is the local optic axis of the layer of interest, it also experiences the same set of rotation to form the binormal vector $B_n$, which is orthogonal to the measured osculating plane and can be computed by the DDG method directly. Hence, the final local optic axis $A_n$ can be obtained by applying a set of 3D rotation operations to the binormal vector $B_n$:

5 $$A_n = R_{{{{\mathrm{n}}}} - 1}\left( { - \delta _{n - 1};\;A_{n - 1}} \right)R_{{{{\mathrm{n}}}} - 2}\left( { - \delta _{n - 2};\;A_{n - 2}} \right) \ldots R_1( - \delta _1;\;A_1)B_n$$

From this equation, the local optic axis can be derived layer by layer using the TNB vectors of the polarization state curve and the 3D rotation matrix of each upper layer.

Finally, we describe how to use the TNB vectors to compute the local phase retardation. Figure 5e shows the actual osculating plane of the n-th layer in Fig. 5d. In this plane, the local phase retardation $\delta _n$ is half the angle that rotates the local input polarization state $S_n^{in}$ about the local optic axis to the local output polarization $S_n^{out}$. That is, $\delta _n = \frac{1}{2}\angle S_n^{in}O^\prime S_n^{out}$. Consider the round-trip measurement, $\phi _{n - 1} = \mathop {\sum}\nolimits_{i = m}^{i = n - 1} {\delta _i = \angle V_{m - 1}O^\prime S_n^{in} = \angle S_n^{in}O^\prime V_{n - 1} = \angle S_n^{out}O^\prime V_n}$, hence, $\delta _n = \frac{1}{2}\angle S_n^{in}O^\prime S_n^{out} = \frac{1}{2}\angle V_{n - 1}O^\prime V_n.$ As mentioned above, there is a rigid rotation of the actual osculating plane to form the measured osculating plane ($P_{m - 1} \ldots P_{n - 1}P_n$) when the light passes through all the upper layers once again. Then we can get $\delta _n = \frac{1}{2}\angle V_{n - 1}O^\prime V_n = \frac{1}{2}\angle P_{n - 1}O^{\prime\prime} P_n$, which is half the angle between the adjacent *N* vectors as shown in Fig. 5f. Thus, the local phase retardation can be expressed as

6 $$\delta _n = \frac{1}{2}arccos\frac{{N_{n - 1} \cdot N_n}}{{\left| {N_{n - 1}} \right|\left| {N_n} \right|}}$$

Equations 5 and 6 show that based on the PSOCT-based polarization state transmission model, the local axis orientation and local phase retardation can be derived using a DDG-based PST method combined with a set of 3D rotation using only one single polarization state.

## Supplementary information

Graphical abstract

Supplemental information

PSOCT imaging of cheek region1

PSOCT imaging of cheek region2

PSOCT imaging of glabella region1

PSOCT imaging of glabella region2

PSOCT imaging of inferior eyelid region2

PSOCT imaging of inferior eyelid region1

PSOCT imaging of lateral eyelid region1

PSOCT imaging of lateral eyelid region2

PSOCT imaging of upper lip region1

PSOCT imaging of upper lip region2
