# Supplementary material for: Polarization sensitive optical coherence tomography with single input for imaging depth-resolved collagen organizations
Source: Light Sci Appl. 2021 Nov 24;10:237. doi: 10.1038/s41377-021-00679-3 (PMC8613400; doi:10.1038/s41377-021-00679-3)

**Short summary**

Discrete differential geometry-based polarization tracing method enables PSOCT imaging of depth-resolved collagen organizations.

**Graphical Abstract**


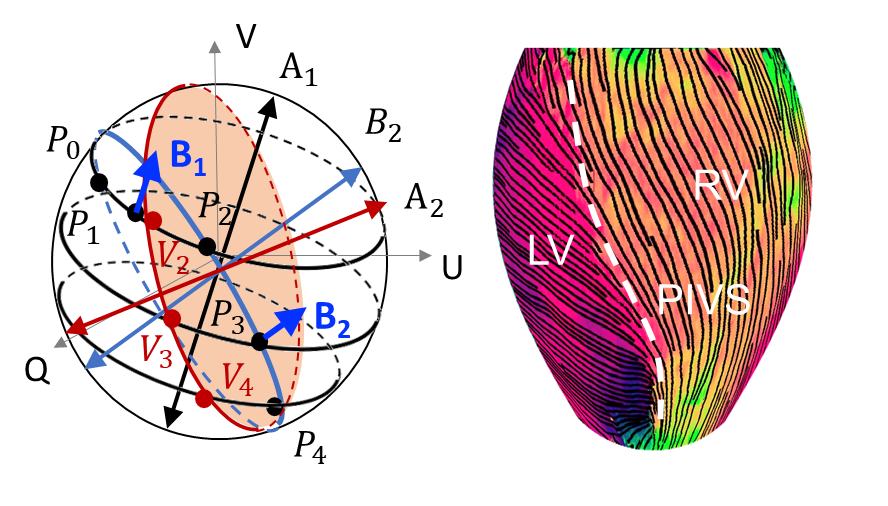

Supplement: Supplementary file 1 — Graphical abstract [file 41377_2021_679_MOESM1_ESM.docx]
