# Supplementary material for: Polarization sensitive optical coherence tomography with single input for imaging depth-resolved collagen organizations
Source: Light Sci Appl. 2021 Nov 24;10:237. doi: 10.1038/s41377-021-00679-3 (PMC8613400; doi:10.1038/s41377-021-00679-3)
Supplement: Supplementary file 2 — Supplemental information [file 41377_2021_679_MOESM2_ESM.docx]

**Title**

**Supplementary Information for polarization sensitive optical coherence tomography with single input for depth-resolved collagen organization imaging**

Peijun Tang^1^, Mitchell A. Kirby^1^, Nhan Le^1^, Yuandong Li^1^, Nicole Zeinstra^1^, G. Nina Lu^2^, Charles E. Murry^1,3,4,5^, Ying Zheng^1,5^, and Ruikang K. Wang^1,6^*

1. Department of Bioengineering, University of Washington, 3720 15th Ave NE, Seattle, WA 98195, USA
2. Department of Otolaryngology- Head and Neck Surgery, Facial Plastic and Reconstructive Surgery, University of Washington, Seattle, WA 98195, USA
3. Department of Pathology, University of Washington, Seattle, WA, 98109, USA
4. Center for Cardiovascular Biology, University of Washington, Seattle, WA, 98109, USA
5. Institute for Stem Cell & Regenerative Medicine, University of Washington, Seattle, WA, 98109, USA
6. Department of Ophthalmology, University of Washington, Seattle, WA 98105, USA

**Running Title:** PSOCT imaging of collagen organization**.**

**Key words:** Polarization sensitive OCT, birefringence, Poincare sphere, optic axis, phase retardation, infarcted heart, skin

*** Address for reprints:**

Ruikang K Wang, PhD

University of Washington,

Box 355061, 3720 15th Ave NE,

Seattle, WA 98195-5061

Phone: 206 616 5025

Fax: 206 685 3300

Email: [wangrk@uw.edu](mailto:wangrk@uw.edu)

**S1. Generalized single input PSOCT system and DDG-based PST method**

A schematic of the generalized single input PSOCT system is shown in Fig. S1. The system used a 100-kHz MEMS-VCSEL swept laser source (SL1310V1-10048, Thorlabs), providing an output power of 25 mW with a central wavelength of 1310 nm and a spectral tuning range of 100 nm. The output of the light source was sent to a polarization controller and became linearly polarized through a polarization beam splitter (PBS 1), and then split into the reference and sample arms through a beam splitter at a split-ratio of 50:50. The reference arm was installed with a quarter-wave plate (QWP) with its axis aligned at 22.5° with reference to the input polarization state, ensuring that the reflected light was coupled equally into the vertical and horizontal channels. The sample arm was equipped with a QWP aligned at 45° with respect to the input polarization state, resulting in a circularly polarized light incident at the sample surface. The light coming back from both the reference and sample arms were recombined and sent to PBS1 and PBS2, respectively, where the interference light was split into horizontal (Channel 1: PD1) and vertical (Channel 2: PD2) components. Balanced detection was used for both vertical and horizontal channels to collect the interference signals, upon which PS-OCT images are reconstructed. The PS-OCT system was controlled using a home-developed LabView (National Instruments) platform which provided flexibility to control scanning patterns based on the applications. The axial resolution was approximately 7.5 μm, in air.


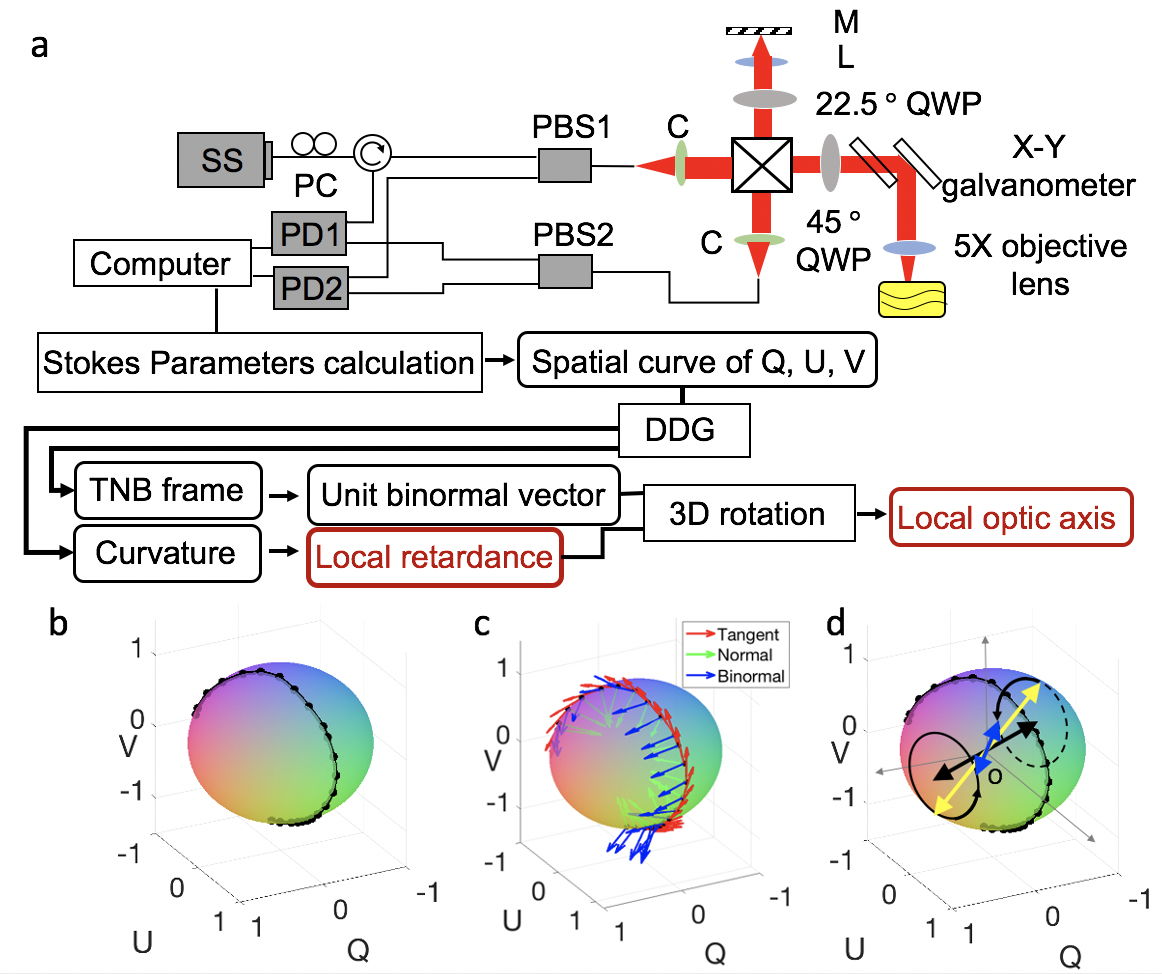


**Fig. S1.** a. Schematic of the fiber-based PS-OCT system. SS: swept source; PC: polarization controller; OC: optical circulator; PBS: polarization beam splitter; QWP: quarter wave plate; PD: photo detector. Below the system schematic is a data processing flow chart of the DDG-based PST method; b. Spatial curve of the output polarization states in a PSOCT A-scan captured from the birefringence phantoms; c. The moving frenet-frame (TNB-frame) of the spatial curve by using the DDG analysis; d**.** 3D rotation to obtain the depth-resolved optic axis.

The flow chart below the system schematic in Fig.S1a illustrates the processing procedures of the DDG-based PST method to derive local phase retardation and optic axis from the PS-OCT measurements. The output polarization states represented by *Q*, *U* and *V* were reconstructed by the Stokes parameters calculation [1] after conventional OCT signal processing [3]. Note that the magnitude of the output polarization state can be decreased by the depolarization [4], making the output polarization states no longer lie at the surface of the Poincare sphere in the Stokes space. To eliminate the effect of the depolarization, all the Stokes vectors of the output polarization states were normalized to 1 before calculating the local phase retardation and local axis orientation. The trajectory of the output polarization states in an A-scan at the Poincare sphere is considered as a spatial curve as shown in Fig. S1b. DDG is applied to this curve to provide the TNB frame of the curve in an A-scan as shown in Fig. S1c. In Fig. S1c, a set of unit tangent vectors $T(n)$, unit normal vectors $N(n)$ and unit binormal vectors $B(n)$ of the curve are presented with red, green and blue arrows respectively. Figure S1d show the 3D geometric relation between the upper optic axes (black arrow), the axis of the measured osculating plane (blue arrow) and the local optic axis (yellow). By using a 3D rotation operation, then the local phase retardations and local axis orientation can be obtained by Eq. 5 and Eq. 6, respectively (See main text).

Because the asymmetry of the optical path in the PSOCT system can cause an overall rotation of the plane that contains all possible optic axes, the vector *An* is a vector in 3D space. After the 3D vector *An* is computed, the effect of the asymmetry of the system can be identified as the *V*-components of all the 3D vectors *An*. Since the vertical (along *z*, or equivalently *V* on the Poincare sphere) component of the vector *An* is introduced by the imaging system and not sensitive to tissue structures, it should be removed. As such, the direction of the vector projected onto the *QU*-plane represents the relative axis orientation of the sample. Note that this is true when the plane of the possible optic axes rotated out of the *QU*-plane with an acute angle. When the plane of the possible optic axes rotated off the *QU*-plane with an obtuse angle, the plane of the possible optic axes will be flipped if directly project it into the *QU*-plane. Hence, a flip operation should be done before finding the absolute axis orientation. To obtain the absolute axis orientation of the sample, we calibrate the system by using a phantom with a known optic axis, and then define this axis as the reference direction (0$^{\circ}$). RGB colors are utilized to code the local axis orientation for display. The projected vector is normalized to a unit vector. Red and green are utilized to code the x and y components of the unit vector that represent the optic axis. Because the *V*-component of *An* is introduced by the system, the color blue (representing the z component of the vectors) is manually set to 0.

**S2. Validation of the DDG-based PST method with birefringence phantom**

We conducted a custom-made birefringent phantom study to validate the proposed DDG-based PST method. The phantom was made of polylactic acid (PLA) 3D printer filament. The PLA exhibits intrinsic homogenous birefringence with its fast optic axis parallel to the filament’s long axis. The phantom was consecutively imaged by the PSOCT system while it was mechanically rotated from 0° to 180° with an increment of ∼10° step. The results are shown in Fig. S2 with a photograph of the sample shown together in Fig. S2a. The 0° was defined as the position when PLA long-axis orientation was parallel to the B-scan direction. Figure S2b shows the cross-sectional structure image of the phantom when it is 90° to the B-scan direction. Without the birefringent information, it’s difficult to determine the axis orientation of the phantom in the structure image. Figure S2c shows the corresponding polarization state image, in which the accumulation effect is presented as multiple band-like patterns. In this case, the axis orientation of the phantom is still hard to be appreciated. Figure S2d shows the local axis orientation image of the sample by using the DDG-based PST method, in which the accumulation effect is removed, and the relatively homogenous depth-resolved axis orientation is retrieved.


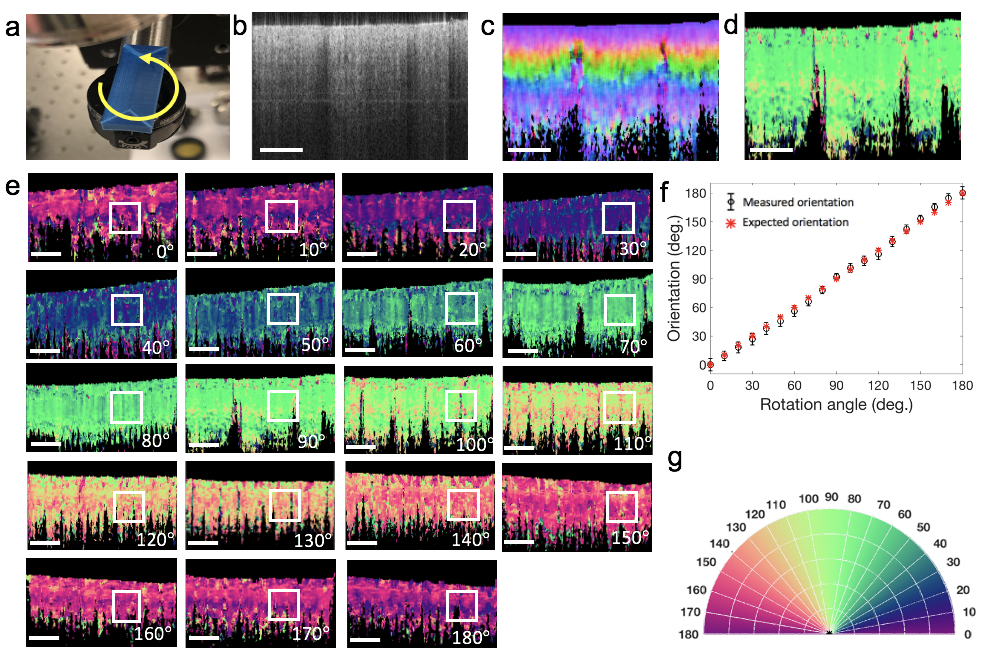


**Fig. S2.** PS-OCT imaging of a PLA filament with a homogenous optic axis rotated from 0° to 180° with a step size of 10°. a. The photograph of the filament and an illustration of the sample rotation. The black arrow indicates the B-scan direction; b. Cross-section structure images of the PLA filament; c. Cross-section polarization state image of the PLA filament, demonstrating the accumulation effect in PSOCT imaging; d. Local axis orientation image of the filament in which the accumulation effect is removed; e. Local orientation images of the filament rotated from 0° to 180° with the rotating angle as shown; f. The scatter plots of the local axis as a function of the rotation angle (from 0° to 180° with a step of 10°). The standard deviations are shown calculated from the region of interest (ROI) marked in the white box in A; g. Colormap used to color-code the orientation images in (d) and (e). The white scale bar is 1 mm.

These results demonstrate that the DDG-based PST method has the ability to reconstruct the depth-resolved axis orientation image. Figure S2e shows cross-sectional local optic axis maps of the phantom sample acquired at different angles, where the images of local axis are relatively uniform at each rotation angle but demonstrate different colors according to the PLA long-axis orientation relative to the B-scan direction. The change trend of the color when the sample was rotated from 0° to 180° is consistent with the colormap in Fig. S2g, demonstrating that the calculated local axis can retrieve the relative sample orientation. The mean and standard deviation of the local optic axis orientation in the region enclosed by the white box in Fig. S2e were calculated and the curves of these values as a function of the rotation degree are plotted in Fig. S2f. In Fig. S1f, the orientation value increases linearly as the rotation angle increases, showing that the calculated local axis provides the relative orientation of the sample.

We next tested the algorithm in a sample with depth-varying optic axis. A phantom specimen was constructed by stacking two pieces of PLA filaments at an angle as shown in Fig. S3c. The upper black sample was positioned at ∼90 deg, and the lower piece was positioned at about 175 deg. In the structure OCT image (Fig. S3a), although two PLA filaments can be differentiated by the scattering signals, the orientation information of the sample is not apparent. Note that the surface of the lower filament is flat, but it appears not leveled in Fig. S3a: the surface of the region covered by the black filament is lower than the other region. This is because the refractive index of the black filament is stronger than the air, hence the corresponding optical path is longer, which makes the portion of blue filament covered by the top black filament appear deeper in the cross-sectional OCT image.

The local optical axis (Fig. S3b) in the two pieces of filaments appears relatively homogeneous with distinct differences in the axis values between them. Both the areas covered by the upper filament (R2 indicated by the black box in Fig. S3b) and the region directly exposed to the probing light (R1) have consistent orientation angles, demonstrating that the proposed method has the ability to derive the local axis orientation from a sample with depth-varying optic axis. 1. To reduce noises, we set a threshold to make a mask to remove the low scattering signals. Some parts between two layers are blank (black regions in Fig. S3b) because corresponding scattering signals (Fig. S3a) in black regions in Fig. S3b are relatively low and hence were masked in this study. In the histogram image (Fig. S3d), the dominant orientations of the two filaments are evaluated at ~90° and ~170° respectively, consistent with the directions of the samples shown in Fig. S3c. This further demonstrates the algorithm’s ability to extract depth-resolved local axis orientation information.


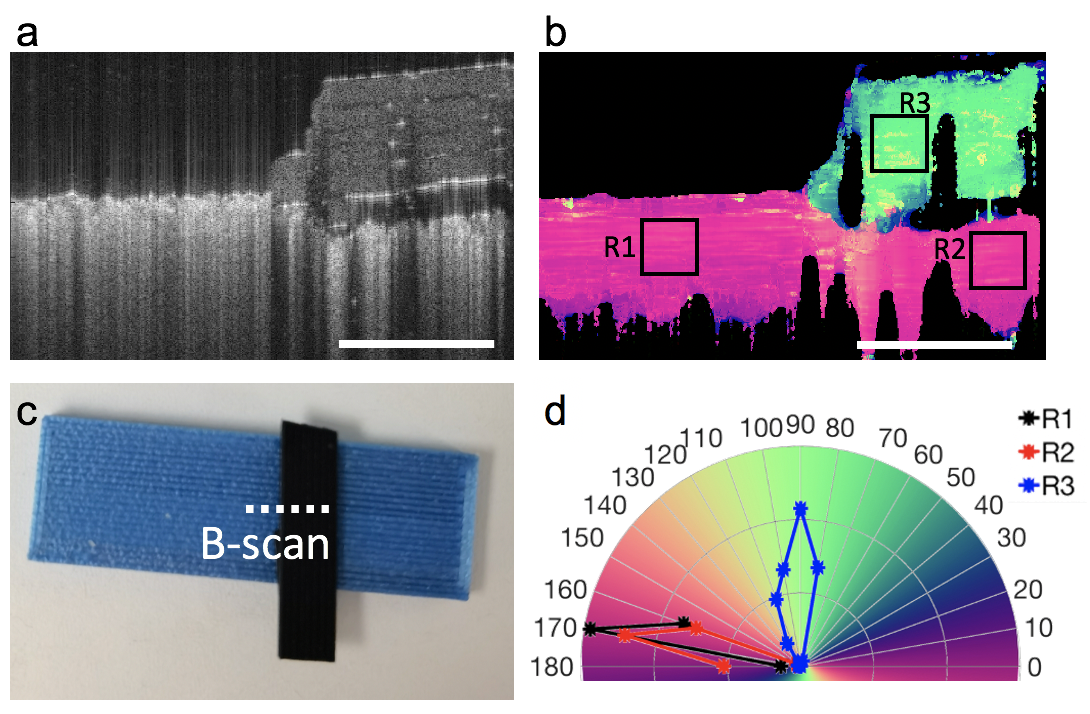


**Fig. S3.** PS-OCT imaging of two PLA filaments stacked at an angle (~90°). a. Cross-sectional of the structure images of the two PLA filaments; b. Local orientation images of the two PLA filaments; c. The photograph of the filaments. White dash line indicates the direction of the B-scan; d. The histogram distributions of orientation obtained from the regions: R1, R2 and R3 indicating by the black boxes in b. The white scale bar is 1 mm.

**S3. Mouse heart imaging**

The myocardial fibers of the whole heart are organized in a helical-like structure to enable its blood pumping capability [5]. Visualizing this exquisite helical-like myocardial architecture of the whole heart is a basic step to investigate the biomechanical dynamic process of the heart beating, which is one of the key parts to elucidate the physiologic cardiac function. To map the myocardial fiber organization of a whole heart, a fresh heart was extracted from a euthanized mouse and placed under the PSOCT system. All experimental procedures in this study were approved by the Institutional Animal Care and Use Committee (IACUC, protocol #2225-04) of the University of Washington and performed in accordance with US NIH Policy on Humane Care and Use of Laboratory Animals. The NA objective lens LSM03 (Thorlabs Inc, USA) was used to provide a FOV of 8 mm x 8 mm with 500 x 500 pixels. The excised whole mouse heart was mounted on a rotational stage using a thin needle. The mouse heart was mechanically rotated from 0° to 360° with an increment of ∼30° step for whole heart imaging. A single-plane en-face image (50 μm below the surface) was used to display macro-level local optical axis. Figure S4a shows the local optic axis orientation at such plane, showing the myofiber architecture of the ventricular wall, for the series of 12 perspective scans. Within each en-face image of the local optical axis, the “stream2” function available in Matlab was used to obtain the “streamline” representation of fiber tracts [6]. A uniform 50 × 50 mesh grid was used as the starting points in streamline calculation of the mouse heart. Then, the 12 en-face images were stitched manually as a whole heart image using photoshop. To retrieve the global structure of the whole heart in 3D space, the whole heart image was wrapped based on cylinder function using matlab. The manual stitching method is not suitable for batch data processing but used in this project for demonstration purposes.


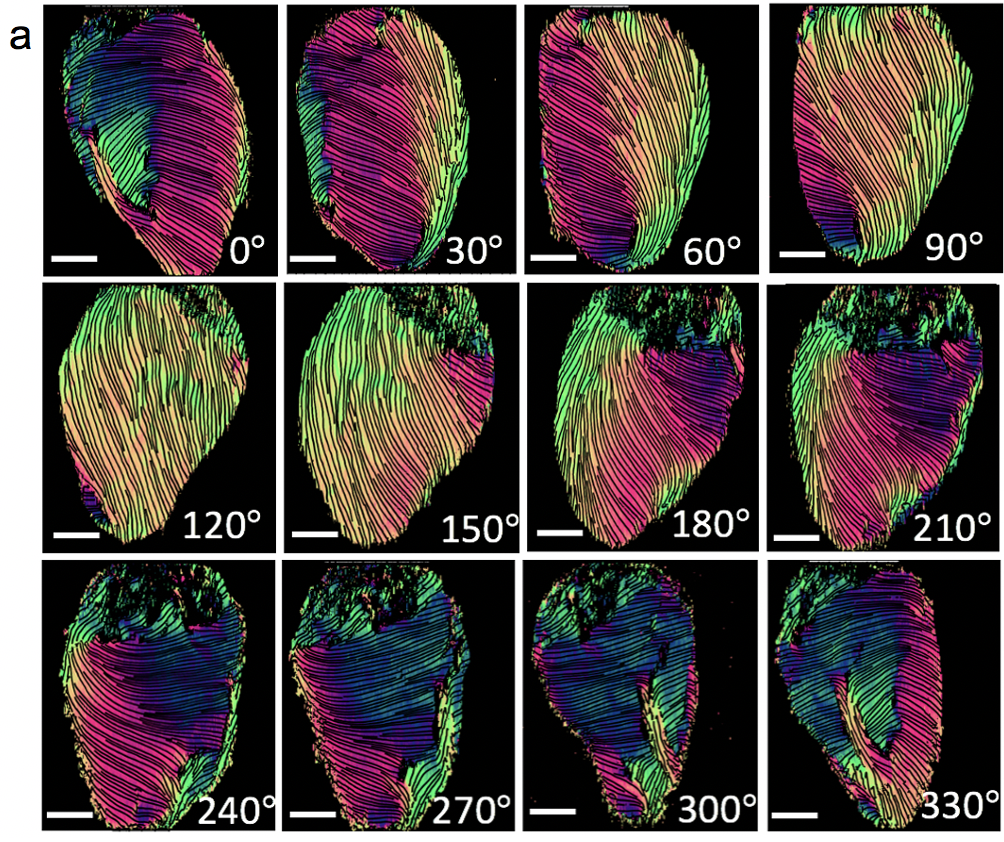


**Fig. S4.** To obtain the whole heart image, the heart was imaged at 12 different perspective angles by rotating it from 0° to 360° with an increment of 30° while with the OCT probe beam fixed in position. a. The surface en-face local axis orientation (OAx) images (50 μm below the heart surface) captured at these 12 angles. anatomical 0° was arbitrarily defined using the aorta as a marker. The colormap is shown in **Fig. S2g**. The scale bar = 1 mm.

**S4. Healthy and infarcted rat heart imaging**

Male Sprague-Dawley rat (approximately 250-300 g, 8 weeks of age) were acquired for imaging of ex vivo heart tissue. All animal procedures were approved by the University of Washington Institutional Animal Care and Use Committee (IACUC, protocol #2225-04) and performed in accordance with US NIH Policy on Humane Care and Use of Laboratory Animals. To compare healthy and infarcted myocardial tissue, one animal was randomly chosen as the healthy sample and another chosen to undergo a thoracotomy surgery for ischemia/reperfusion of the left anterior descending coronary artery to create a myocardial infarction.

For this surgery, rats were anesthetized with an intraperitoneal (IP) injection of 68.2 mg/kg ketamine and 4.4 mg$\cdot$kg^-1^ xylazine. A second dose of full-strength ketamine/xylazine followed by additional ketamine boosts (20  mg$\cdot$kg^-1^, administered as needed) were used to maintain a surgical plane of anesthesia. During the procedure, the rats were intubated, mechanically ventilated, and maintained on a water-circulating head pad. Core body temperature was monitored at regular intervals and maintained at 37 °C. To induce myocardial infarction, the heart was exposed, and the left anterior descending coronary artery was ligated and occluded for 60 min, followed by reperfusion and aseptic chest closure. Sustained release buprenorphine (1 mg$\cdot$kg^-1^) was administered following the surgery to provide analgesia for at least 2 days. Rats were closely monitored for 48 hours to provide post-operative care to ensure and maintain animal health and comfort as outlined in the IACUC protocol.

At 4 weeks post-infarct, rats were euthanized with a chemical overdose of pentobarbital/phenytoin solution (Beuthanasia; 1.5 mL IP injection). Once the animals achieved deep anesthesia while the heart was still beating, the chest was opened, and 50 U Heparin was intravenously infused via the inferior vena cava and allowed to circulate for 1–2 min to prevent thrombosis in the coronary vessels. Intravenous infusion with supersaturated potassium chloride (KCl) was then used to arrest the heart in diastole followed immediately with excision of the heart. The aorta was cannulated followed by retrograde perfusion with a vasodilator buffer (PBS containing 4 mg$\cdot$L^-1^ Papaverin and 1 g$\cdot$L^-1^ adenosine) followed by 4% paraformaldehyde perfusion for 10 min. Perfusion pressure was maintained at ~100 mm Hg. After perfusion fixation, the hearts were transferred to fresh fixative overnight at 4 °C. After overnight fixation, the cannulated hearts were transferred to PBS buffer and then transferred on ice for PS-OCT imaging.

Following PS-OCT imaging, the rat hearts were sliced into 2 mm-thick sections from the apex for paraffin processing and embedding [7, 8]. 4 µm sections were cut and stained for picrosirius red/fast green to visualize the collagenous/infarcted regions in red and healthy tissue in green.

Histology staining of the healthy and infarcted rat hearts are provided to demonstrate that the infarction procedure was successfully applied to the rat heart. Figure S5a shows the histology staining slice of the healthy rat heart. Homogenous green color is observed across the whole slice, indicating that there is no infarction in this sample. On the contrary, red color is found in the deeper regions in Fig. S5b demonstrating that infarction occurs in this heart.

**
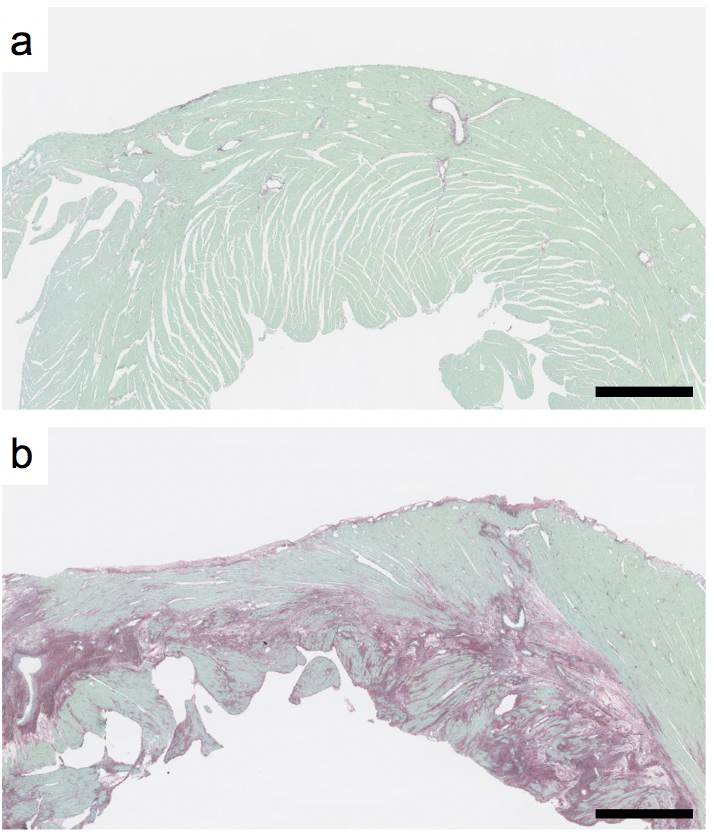
**

**Fig. S5.** Histology staining slices of a. the healthy rat heart b. the infarcted rat heart. The slices are at approximately 6 mm from the apex. Slices are stained with picrosirius red/fast green. Collagenous/infarcted regions stain red, and healthy tissue stains green. The scale bar represents 1 mm.

**S5. In-vivo collagen organization imaging of human facial skin**

**Human facial skin measurement protocol**

Multiple anatomical locations were imaged on the selected regions of facial skin in a healthy volunteer (29 years, female, Asian). The use of OCT to image body skin was approved by the University of Washington institutional review board (IRB00000889). Informed consent was obtained from the volunteer. The selected regions of interest were typical regions in the aesthetic units: 1. glabella, 2. lateral eyelid, 3. cheek 4. inferior eyelid and 5. skin around the upper lip as shown in Fig. 3a (see main text). The Relaxed Skin Tension Lines (RSTLs) [9,10] of these regions were estimated and displayed on the photograph for reference [11,12]. Two OCT scanning patterns were used for imaging: high-lateral resolution scanning (11 μm) with a field of view (FOV) of 2 mm x 2 mm, and relatively low-lateral resolution scanning (22 μm) with a FOV of 6 mm x 6 mm. For each scanning pattern, the OCT beam sampling density was 500 x 500 pixels, i.e. the fast B-scan axis consists of 500 A-scans and the slow axis consists of 500 B-scans. A single-plane en-face image (50 μm below the surface) was used to display local optical axis.

Multiple anatomical locations (defined according to aesthetic units shown in Fig. 3a, please see main text) were imaged on the face of the healthy volunteer. Figures S6-S9 show the in vivo PSOCT and OCT results taken from glabella, lateral eyelid, inferior eyelid and upper lip of the healthy human skin respectively at three certain depths (105 μm, 175 μm and 245 μm).

The resulted 3D PSOCT results for these scans to show the axis orientations of the skin at all depths are provided as supplementary video files for more information. The video files are named as: glabella (LSM 03).mov, glabella (LSM 02).mov, lateral eyelid (LSM 03) .mov, lateral eyelid (LSM 02) .mov, inferior eyelid (LSM 03) .mov, inferior eyelid (LSM 02) .mov, cheek (LSM 03) .mov, cheek (LSM 02) .mov, upper lip (LSM 03) .mov and upper lip (LSM 02) .mov, respectively.

Specifically, Fig. S6a and S6c show the en-face local axis orientation (OAx) images and the corresponding en-face OCT intensity images of the glabella skin at three certain depths (105 μm, 175 μm and 245 μm) using LSM03 and LSM02 respectively. Figure S6b show the 3D polarization state image of the glabella skin. Figure S6d shows the cross-sectional OAx images at the area indicated by the white dash line (upper and bottom figure corresponds to Fig. S6a and S6c respectively). The histogram distributions of orientation obtained from the en-face OAx slices using LSM03 in Fig. S6a at three different depths (105 μm, 175 μm and 245 μm) are shown in Fig. S6e. These results show the depth-resolved collagen architecture of the glabella skin.


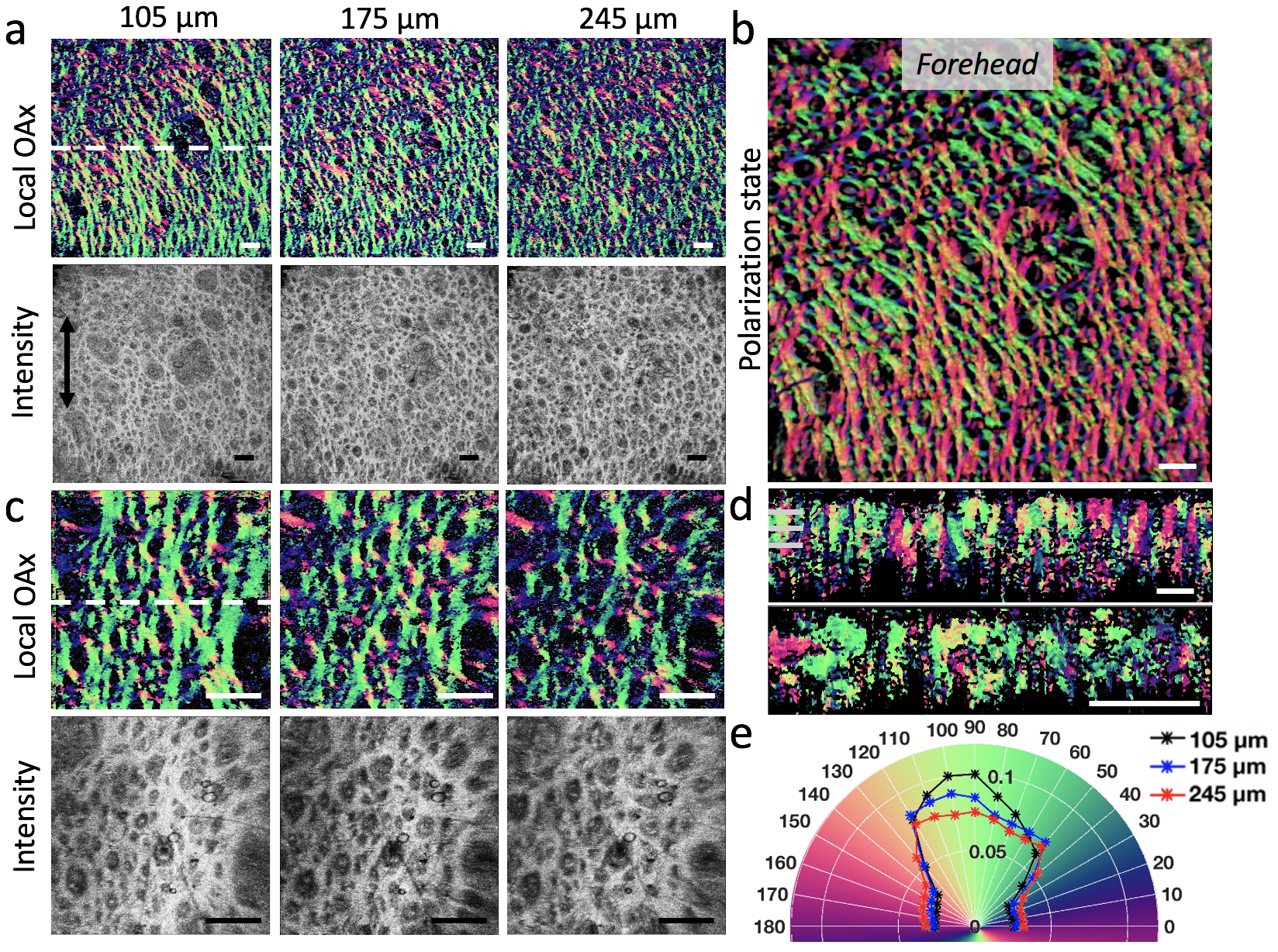


**Fig. S6.** PSOCT results of the human glabella skin (measurement point 1 in Fig. 3a). (a, c) The en-face local axis orientation (OAx) images and the corresponding en-face OCT intensity images at three certain depths (105 μm, 175 μm and 245 μm) using LSM03 and LSM02 respectively; (b) 3D polarization state image; (d) Cross-sectional OAx images at the area indicated by the white dash line (upper and bottom figure corresponds to (a, c) respectively); (e) The histogram distributions of orientation obtained from the en-face OAx slices using LSM03(a) at three different depths (105 μm, 175 μm and 245 μm) respectively. The scale bar = 500 μm. Please also see the PSOCT movies showing axis orientations at all depths: glabella (LSM 03).mov and glabella (LSM 02).mov

Fig. S7a and S7c show the en-face local axis orientation (OAx) images and the corresponding en-face OCT intensity images of the human lateral eyelid at three certain depths (105 μm, 175 μm and 245 μm) using LSM03 and LSM02 respectively. Figure S7b show the 3D polarization state image of the human lateral eyelid. Figure S7d shows the cross-sectional OAx images at the area indicated by the white dash line (upper and bottom figure corresponds to Fig. S7a and S7c respectively). The histogram distributions of orientation obtained from the en-face OAx slices using LSM03 in Fig. S7a at three different depths (105 μm, 175 μm and 245 μm) are shown in Fig. S7e. These results show the depth-resolved collagen architecture of the human lateral eyelid.


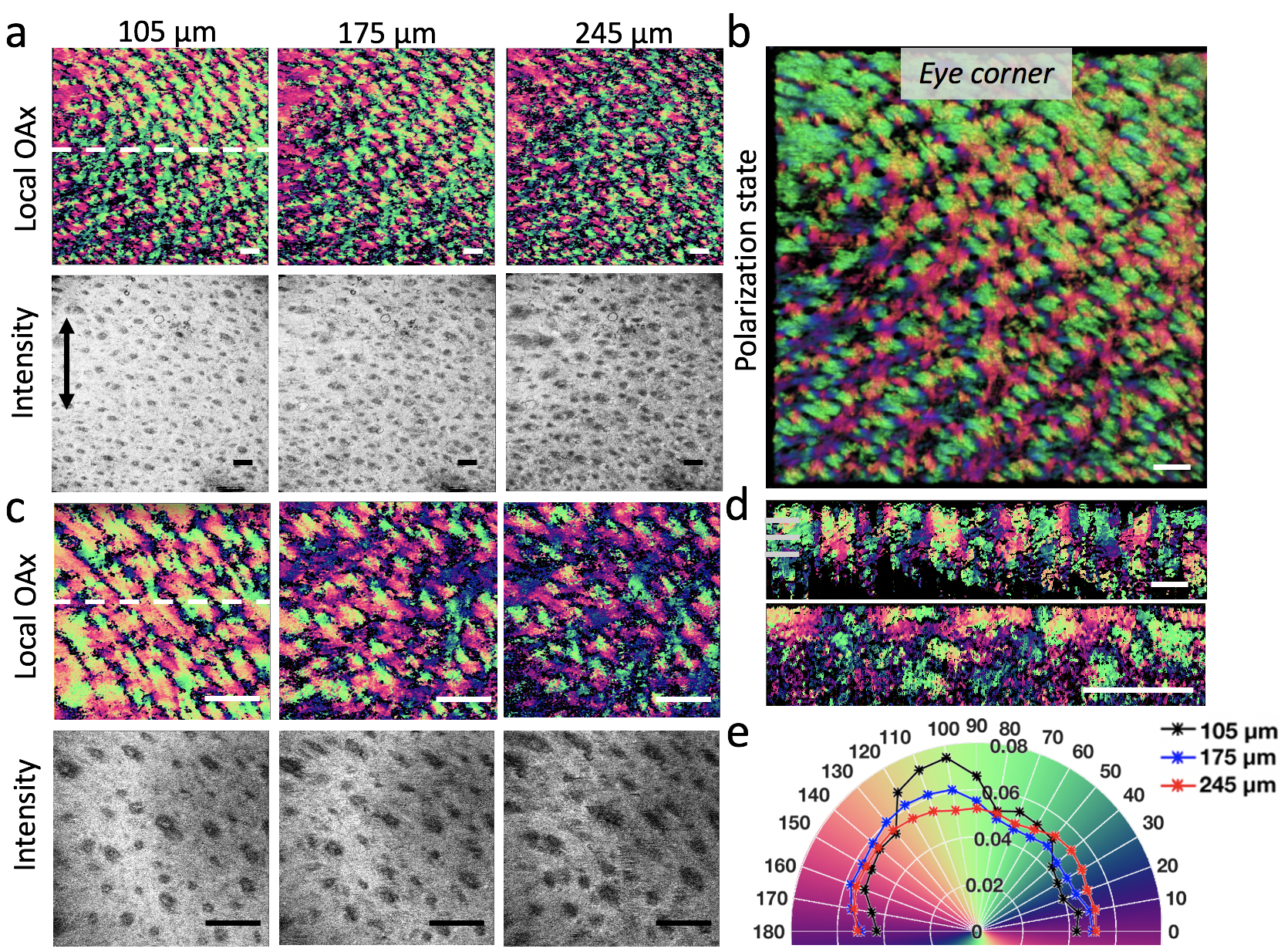


**Fig. S7.** PSOCT results of the human lateral eyelid (measurement point 2 in Fig. 3a). (a, c) The en-face local axis orientation (OAx) images and the corresponding en-face OCT intensity images at three certain depths (105 μm, 175 μm and 245 μm) using LSM03 and LSM02 respectively; (b) 3D polarization state image; (d) Cross-sectional OAx images at the area indicated by the white dash line (upper and bottom figure corresponds to (a, c) respectively); (e) The histogram distributions of orientation obtained from the en-face OAx slices using LSM03(a) at three different depths (105 μm, 175 μm and 245 μm) respectively. The scale bar = 500 μm. Please also see the PSOCT movies showing axis orientations at all depths: lateral eyelid (LSM 03).mov and lateral eyelid (LSM 02).mov


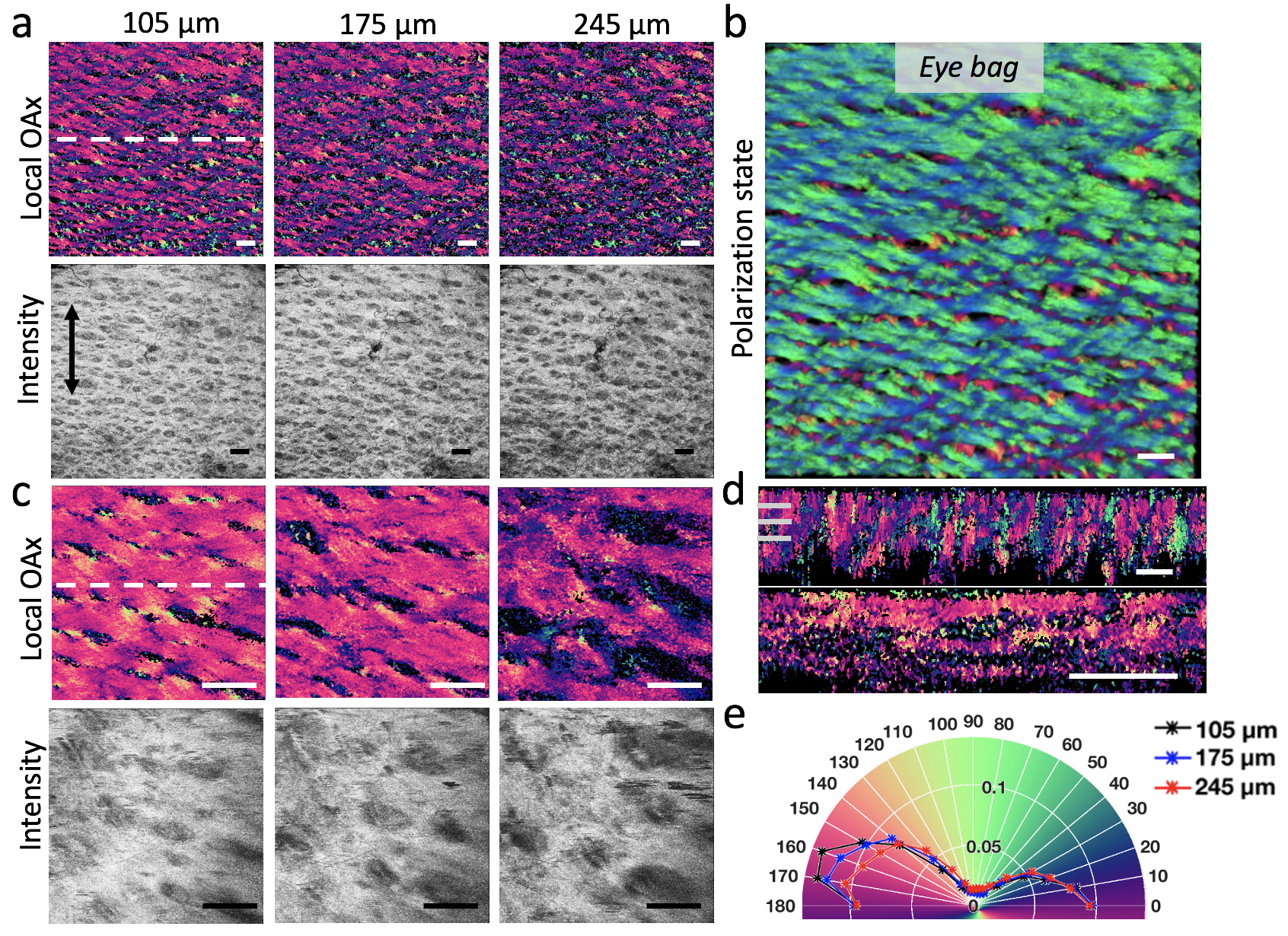


**Fig. S8.** PSOCT results of the human inferior eyelid skin (measurement point 3 in Fig. 3a). (a, c) The en-face local axis orientation (OAx) images and the corresponding en-face OCT intensity images at three certain depths (105 μm, 175 μm and 245 μm) using LSM03 and LSM02 respectively; (b) 3D polarization state image; (d) Cross-sectional OAx images at the area indicated by the white dash line (upper and bottom figure corresponds to (a, c) respectively); (e) The histogram distributions of orientation obtained from the en-face OAx slices using LSM03(a) at three different depths (105 μm, 175 μm and 245 μm) respectively. The scale bar = 500 μm. Please also see the PSOCT movies showing axis orientations at all depths: inferior eyelid (LSM 03).mov and inferior eyelid (LSM 02).mov

Fig. S8a and S8c show the en-face local axis orientation (OAx) images and the corresponding en-face OCT intensity images of the human inferior eyelid skin at three certain depths (105 μm, 175 μm and 245 μm) using LSM03 and LSM02 respectively. Figure S8b show the 3D polarization state image of the human inferior eyelid skin. Figure S8d shows the cross-sectional OAx images at the area indicated by the white dash line (upper and bottom figure corresponds to Fig. S8a and S8c respectively). The histogram distributions of orientation obtained from the en-face OAx slices using LSM03 in Fig. S8a at three different depths (105 μm, 175 μm and 245 μm) are shown in Fig. S8e. These results show the depth-resolved collagen architecture of the human inferior eyelid skin.

**
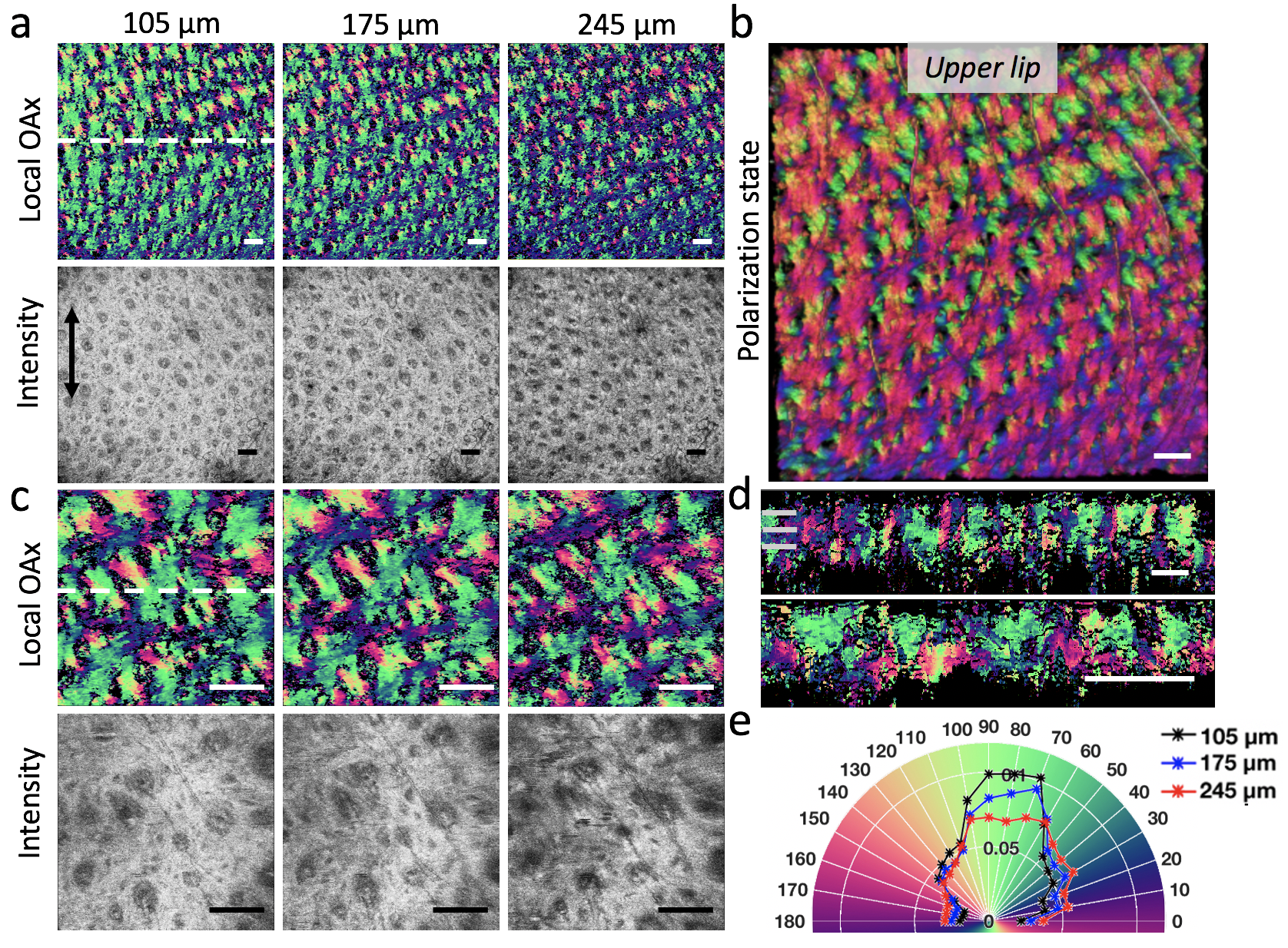
**

**Fig. S9.** PSOCT results of the human upper lip skin (measurement point 5 in Fig. 3a). (a, c) The en-face local axis orientation (OAx) images and the corresponding en-face OCT intensity images at three certain depths (105 μm, 175 μm and 245 μm) using LSM03 and LSM02 respectively; (b) 3D polarization state image; (d) Cross-sectional OAx images at the area indicated by the white dash line (upper and bottom figure corresponds to (a, c) respectively); (e) The histogram distributions of orientation obtained from the en-face OAx slices using LSM03(a) at three different depths (105 μm, 245 μm and 315 μm) respectively. The scale bar = 500 μm. Please also see the PSOCT movies showing axis orientations at all depths: upper lip (LSM 03).mov and upper lip (LSM 02).mov

Fig. S9a and S9c show the en-face local axis orientation (OAx) images and the corresponding en-face OCT intensity images of the human upper lip skin at three certain depths (105 μm, 175 μm and 245 μm) using LSM03 and LSM02 respectively. Figure S9b show the 3D polarization state image of the human upper lip skin. Figure S9d shows the cross-sectional OAx images at the area indicated by the white dash line (upper and bottom figure corresponds to Fig. S9a and S9c respectively). The histogram distributions of orientation obtained from the en-face OAx slices using LSM03 in Fig. S9a at three different depths (105 μm, 175 μm and 245 μm) are shown in Fig. S9e. These results show the depth-resolved collagen architecture of the human upper lip skin.

**S6. Advantage of measuring local axis orientation in 3D Stoke space**

Due to the asymmetric property of the imaging system, the optic axis to be measured becomes a 3D vector in the Stokes space rather than a scalar constrained within the *QU*-plane [13]. Hence, computing the 3D local axis orientation in the Stokes space would provide a higher accuracy.

To show that it is necessary to obtain the local axis orientation in 3D Stokes space which can deliver better performance in the imaging, we performed a comparison study. In the comparison, we evaluated the performances delivered by the previous algorithm that derive the local optic axis in *QU*-plane [14] (Evaluation 1) and by our algorithm (Evaluation 2), respectively. We used the same data set acquired from the facial cheek skin in the evaluations. The Evaluation 1 assumes that the effect of the asymmetry of the system can be ignored, and the 2D measurement of the local axis orientation is sufficient to derive the local optic axis. Under this assumption, the measured local axis orientation is considered to be a scalar constrained within the *QU*-plane.

The Evaluation 2 assumes that the measured local axis orientation is a 3D vector due to the asymmetrical property of the system, which is proposed in our study. In this case, the 3D optic axis of each layer was computed and used to derive the local optic axis layer by layer until the maximum depth has reached as described in our method. Note that in the final step of our method, there has an operation that sets the *V*-component of all final local optic axes to zero. This operation is to eliminate the effect induced by the system asymmetry. Different from the Evaluation 1, this operation was only executed at the final step after all the computations of the 3D local optic axes have been completed in 3D space.

The null hypothesis here is that if the effect of the asymmetry of the system can be ignored in the evaluation of the local optic axis, then the resulting optic axis delivered by the above two evaluations would be similar.

Figure S10 below shows the en-face local phase retardation and local axis orientation images resulted from the two Evaluations, respectively. The enface images at two depths (175 μm and 315 μm) are selected to demonstrate the noise and error accumulation effect over depth. The first raw (Figs. S10a-d) show the results from the Evaluation 1 (based on the 2D measurement), whereas the second raw (Figs. S10e-h) show the results from the Evaluation 2 (based on the 3D model described in our paper). By a quick eyeballing, the testing hypothesis as stated above fails. It is also clear that the results delivered by the 3D model have much higher contrast and signal to noise ratio, demonstrating the advantages of the use of the 3D model to derive the optic axis and phase retardation.

Specifically, in the local phase retardation results (Figs. S10a, S10c, S10e and S10g), depth-resolved collagen organization can be visualized by both the methods. However, part of the signals generated by the collagen (as indicated by the yellow arrows and boxes) are lost in the 2D model (Fig. S10a and S10c) but appear clearly in the results from our method (Fig. S10e and S10g). The low contrast and noisy appearance in the images of the 2D model are largely due to the errors introduced by the consideration of 2D scalar optic axis at each step of the evaluation (and this error accumulates and propagates to the next level when the optic axis and phase retardation are computed at the deeper depths). This observation demonstrates that ignoring the *V*-component of the measured optic axis can lead to non-negligible information loss.


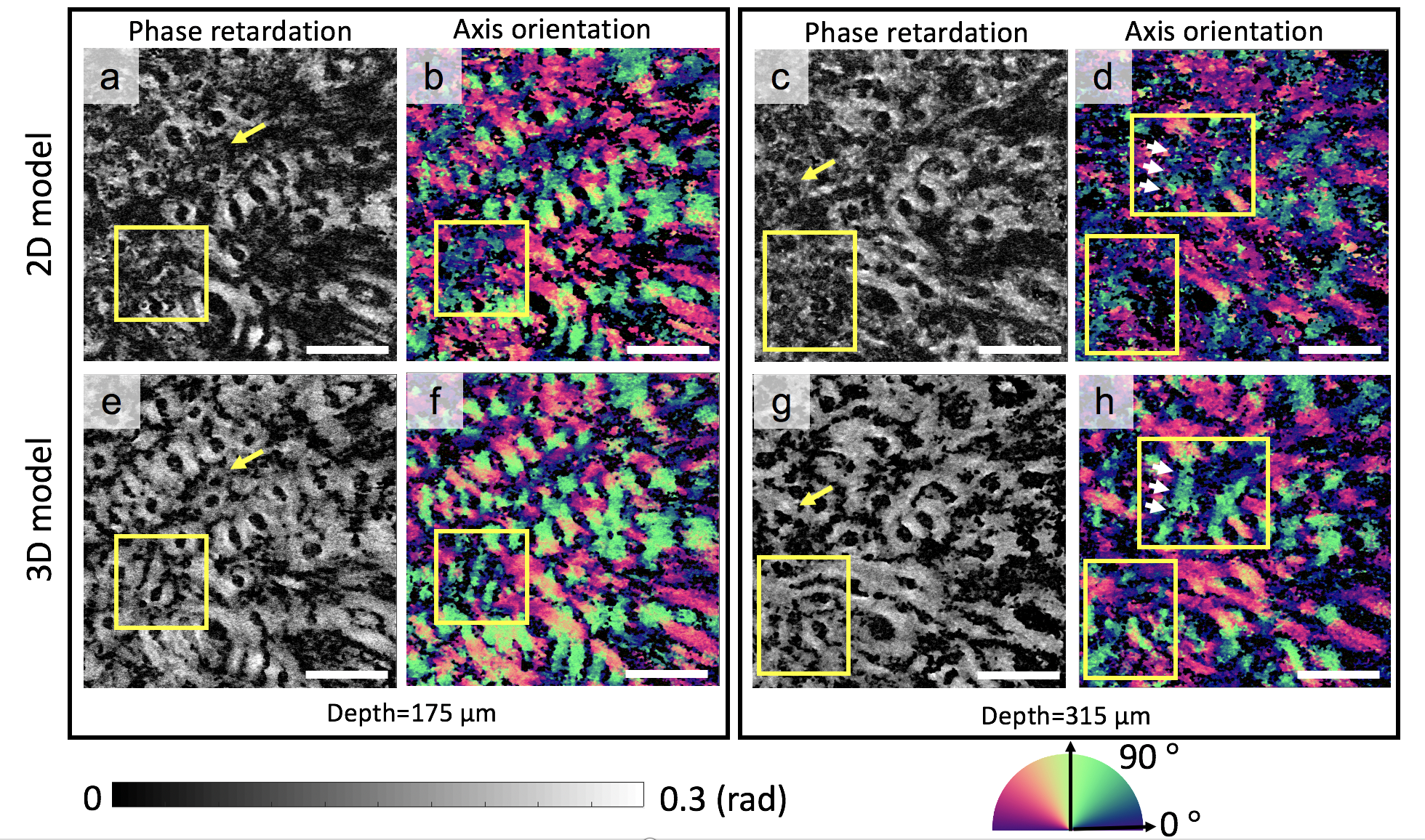


**Figure S10**. En-face local phase retardation and local axis orientation images resulted from the two Evaluations: i.e., 2D and 3D models, respectively. Results shown are obtained from a selected region of in-human facial cheek skin (marked as red square in Fig. 3a in the paper). (a-d) The en-face local phase retardation (a, c) and local axis orientation (b, d) images computed based on the 2D model. (e-h) The en-face local phase retardation (e, g) and local axis orientation (f, h) images computed based on the 3D model. (a, b, e and f) and (c, d, g and h) are the en-face images selected at 175 μm and 315 μm respectively. The scale bar = 500 μm. The color maps to display the phase retardation and optic axis images are shown at the bottom, respectively.

In the local axis orientation results selected at the depth of 175 microns (Fig. S10b and S10f), the orientation values of the collagen fiber bundles are relatively consistent with its corresponding morphologic direction in both groups of results, demonstrating that both computations can derive the local axis orientation of the sample. However, in the region with phase retardation information loss (as indicated by the yellow boxes), the orientation values of the collagen are inexplainable in Fig. S10b while the orientation values in Fig. S10f can clearly present the direction of the collagen, indicating that using the 3D model to compute the local axis orientation can provide a higher accuracy. The inaccurate measurement of the local axis orientation of the superficial layer will severely affect the computation of the local axis orientation in the deeper layer because the errors can propagate and accumulate over depth.

Because of accumulation effect, the impact of the dimension reduction on the final result would be more severe in the deeper depths. Figures S10d and S10h show the en-face local axis orientation images at 315 μm below the skin surface. The collagen organization can still be clearly differentiated in Fig. S10h with high contrast and SNR, whereas it is not in Fig. S10d. Moreover, the orientation values of a collagen bundle as indicated by the white arrows show an obvious difference in these two images: the mean orientation values of this collagen bundle are ~35° and ~73° in Fig. S10d and Fig. S10h, respectively. Referring to the morphologic direction of the collagen, the orientation value in Fig. S10h is more accurate than that in the Fig. S10d. These results demonstrate that the bias of the orientation induced by the dimension reduction will be accumulated over depth. Even the bias is small in the surface layer, it can still generate non-negligible effect to the measurement in the deeper layer.

In summary, it is necessary to compute the local axis orientation in 3D space due to the asymmetry property of the system. Note that in clinical translation, the system asymmetry property is environment dependent, therefore, we believe that our proposed method with a single input facilitates the clinical translation.

**Supplementary information accompanies the manuscript on the Light: Science & Applications website (http://www.nature.com/lsa)**

**References**

1. Tang, P. J., Xu, J. J. & Wang, R. K. Imaging and visualization of the polarization state of the probing beam in polarization-sensitive optical coherence tomography. Applied Physics Letters 113, 231101 (2018).
2. Tang, P.J. & Wang, R.K. Polarization state tracing method to map local birefringent property in sample using polarization sensitive optical coherence tomography. Biomedical Optics Express. **11**, 6852-6863 (2020).
3. Wang, R. K. & Ma, Z. Z. A practical approach to eliminate autocorrelation artefacts for volume-rate spectral domain optical coherence tomography. *Physics in Medicine & Biology* **51**, 3231 (2005).
4. Ortega-Quijano, N., Marvdashti, T. & Ellerbee Bowden, A. K. Enhanced depolarization contrast in polarization-sensitive optical coherence tomography. *Optics Letters* **41**, 2350-2353 (2016).
5. Trainini, J. C. *et al.* New Research in The Anatomy Of The Myocardium. *Trends in Anatomy & Physiology* **2**, 1-12 (2019).
6. Fan, C. M. & Yao, G. Imaging myocardial fiber orientation using polarization sensitive optical coherence tomography. Biomedical Optics Express 4, 460-465 (2013).
7. Gerbin, K. A. *et al.* Enhanced Electrical Integration of Engineered Human Myocardium via Intramyocardial versus Epicardial Delivery in Infarcted Rat Hearts. *PLoS One* **10**, e0131446 (2015).
8. Laflamme, M. A. *et al.* Cardiomyocytes derived from human embryonic stem cells in pro-survival factors enhance function of infarcted rat hearts. *Nature Biotechnology* **25**, 1015-1024 (2007).
9. Piérard, G. & Lapière, C. Microanatomy of the dermis in relation to relaxed skin tension lines and Langer's lines. *The American Journal of Dermatopathology* **9**, 219-224 (1987).
10. Borges, A. F. Relaxed Skin Tension Lines. Dermatologic Clinics **7**, 169-178 (1989).
11. Langer, A. K. On the anatomy and physiology of the skin: I. The cleavability of the cutis. *British Journal of Plastic Surgery* **31**, 3-8 (1978).
12. Alhamdi, A. Facial Skin Lines. *Iraqi Journal of medical sciences* **13**, 103-107 (2015).
13. Park, B. *et al.* Optic axis determination accuracy for fiber-based polarization-sensitive optical coherence tomography. *Optics Letters* **30**, 2587-2589 (2005).
14. Fan, C. M. & Yao, G. Mapping local optical axis in birefringent samples using polarization-sensitive optical coherence tomography. Journal of Biomedical Optics 17, 110501 (2012).
